# Inactivation of a Human Kinetochore by Specific Targeting of Chromatin Modifiers

**DOI:** 10.1016/j.devcel.2008.02.001

**Published:** 2008-04-15

**Authors:** Megumi Nakano, Stefano Cardinale, Vladimir N. Noskov, Reto Gassmann, Paola Vagnarelli, Stefanie Kandels-Lewis, Vladimir Larionov, William C. Earnshaw, Hiroshi Masumoto

**Affiliations:** 1 Laboratory of Molecular Pharmacology, National Cancer Institute, National Institutes of Health, Building 37, Room 5040, 9000 Rockville Pike, Bethesda, MD 20892, USA; 2 Wellcome Trust Centre for Cell Biology, University of Edinburgh, King's Buildings, Mayfield Road, Edinburgh EH9 3JR, Scotland, United Kingdom

**Keywords:** CELLBIO, DNA

## Abstract

We have used a human artificial chromosome (HAC) to manipulate the epigenetic state of chromatin within an active kinetochore. The HAC has a dimeric α-satellite repeat containing one natural monomer with a CENP-B binding site, and one completely artificial synthetic monomer with the CENP-B box replaced by a tetracycline operator (tetO). This HAC exhibits normal kinetochore protein composition and mitotic stability. Targeting of several tet-repressor (tetR) fusions into the centromere had no effect on kinetochore function. However, altering the chromatin state to a more open configuration with the tTA transcriptional activator or to a more closed state with the tTS transcription silencer caused missegregation and loss of the HAC. tTS binding caused the loss of CENP-A, CENP-B, CENP-C, and H3K4me2 from the centromere accompanied by an accumulation of histone H3K9me3. Our results reveal that a dynamic balance between centromeric chromatin and heterochromatin is essential for vertebrate kinetochore activity.

## Introduction

In budding yeast, kinetochore assembly begins with the recognition of key DNA sequences by sequence-specific DNA-binding proteins. The cloning of yeast centromere DNA and the subsequent development of artificial chromosomes (YACs; Murray and Szostak, 1983) allowed detailed characterization of the protein and DNA components, so that the budding yeast has the best-characterized of all kinetochores (Lechner and Carbon, 1991; Doheny et al., 1993). Other kinetochores remain less well understood, partly because as first observed in *S. pombe* (Steiner and Clarke, 1994; Ekwall et al., 1997), epigenetic effects play a major role in centromere specification (Earnshaw and Migeon, 1985; Alonso et al., 2003; Chueh et al., 2005).

The isolation of human artificial chromosomes (HACs) offers a powerful way to study vertebrate kinetochore assembly. HACs have been obtained in HT1080 human fibrosarcoma cells using regular arrays of alphoid DNA derived from human centromeres (Harrington et al., 1997; Ikeno et al., 1998; Henning et al., 1999; Ebersole et al., 2000; Grimes et al., 2001; Mejia et al., 2001). HAC kinetochores accurately mimic the structure and mitotic behavior of endogenous kinetochores (Tsuduki et al., 2006). The cloned alphoid DNA assembles into kinetochore-specific chromatin with CENP-A (Sullivan and Karpen, 2001; Black et al., 2004) and CENP-C (Saitoh et al., 1992), and carries core histone modifications characteristic of centromeric chromatin (Nakashima et al., 2005).

A specialized structure of kinetochore chromatin was first shown for the central core of the *S. pombe* kinetochore (Takahashi et al., 1992; Saitoh et al., 1997), which is flanked by heterochromatin-like regions (Allshire et al., 1994; Partridge et al., 2000). More recently, a novel form of chromatin termed “centromere chromatin” in which CENP-A clusters are interspersed with clusters of dimethylated histone H3 Lys4 (H3K4me2) has been proposed for human, *D. melanogaster*, and rice centromeres (Blower et al., 2002; Nagaki et al., 2004; Sullivan and Karpen, 2004), as well as a human neocentromeres (Chueh et al., 2005).

The goal of the present study was to establish a system permitting manipulation of the epigenetic status of a single kinetochore in living cells. Prior attempts to manipulate centromere chromatin have involved treatment of cells with chemical inhibitors or protein overexpression and/or knockdowns. Such treatments affect all centromeres, and may alter additional aspects of cellular physiology as well. Here, we describe an HAC in which the underlying alphoid DNA repeat contains a tetracycline repressor (tetR) binding site (tetO). This enables us to target any desired protein into the active kinetochore of the alphoid^tetO^ HAC as a tetR fusion protein. Our results reveal that kinetochore activity appears to depend upon the status of the underlying chromatin. We show that although introducing a transcriptional activator into alphoid DNA can lead to centromere inactivation, surprisingly, introducing a transcriptional repressor into the centromere has a much stronger effect, leading to the rapid and quantitative loss of kinetochore function.

## Results

### HAC Formation Using a Synthetic Alphoid DNA Dimer

**Figure 1:**
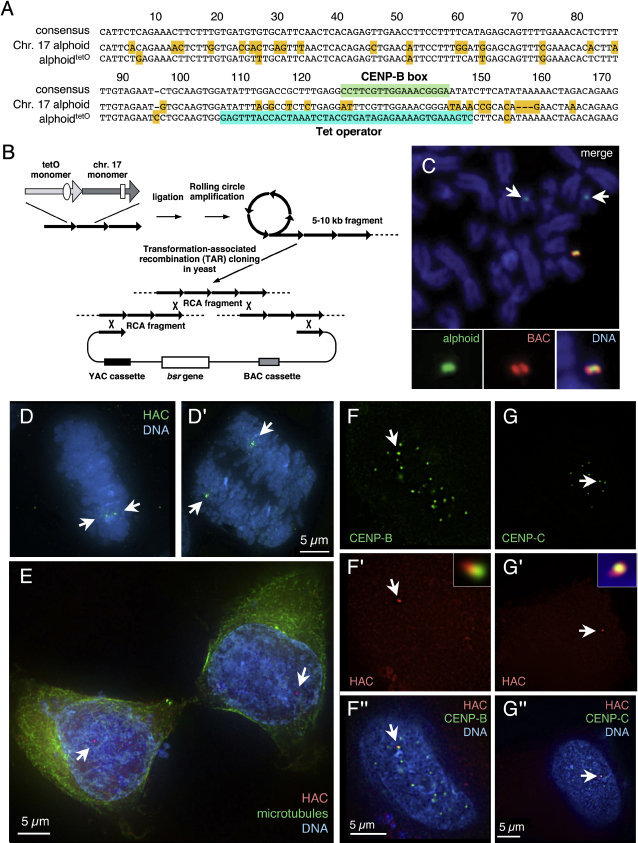
Isolation of the Alphoid^tetO^ HAC (A) Sequence comparison between the alphoid monomers used for the alphoid^tetO^ DNA array and the alphoid consensus. One monomer of the alphoid^tetO^ dimer is derived from a chromosome 17 alphoid type I 16-mer unit and contains a CENP-B box (shaded green). The second monomer is a wholly synthetic sequence derived from the Choo consensus (Choo et al., 1991), with sequences corresponding to the CENP-B box replaced by a 42 bp tetO motif (shaded blue). Other bases that differ from the consensus are shaded in yellow. (B) Diagram showing the construction of the alphoid^tetO^ BAC by rolling circle amplification (RCA) in vitro and transformation-associated recombination (TAR) cloning in yeast cells. This yielded BAC clone, BAC32-2-mer(tetO), containing 50 kb of the alphoid^tetO^ dimer DNA. (C) FISH analysis of a metaphase cell chromosome spread containing the alphoid^tetO^ HAC (AB2.2.18.21) with alphoid^tetO^ (green) and BAC vector probes (red). Chromosomes were counterstained with DAPI (blue in merged panels). Endogenous chromosome 17 centromeres were also detected by the alphoid^tetO^ probe (arrows in upper panel). (D and D′) FISH on AB 2.2.18.21 cells in metaphase (D) and anaphase (D′). Arrows indicate the HAC undergoing normal segregation. (E) Cell in cytokinesis transfected with mRFP-TetR (red) and stained with anti-tubulin antibody (green) and DAPI (blue). mRFP-TetR binds to the HAC (arrows), in the two daughter cells. (F and G) mRFP-TetR binds to the alphoid^tetO^ HAC in vivo where it colocalizes (arrows) with CENP-B (F), CENP-C (G) (both green), and DNA ([F″ and G″], blue). Size bars in (D–G) = 5 μm.

To obtain a human artificial chromosome (HAC) suitable for manipulation of the epigenetic status of the kinetochore, we designed a novel artificial alphoid^tetO^ dimer with one monomer from a chromosome 17 alphoid 16-mer higher order repeat (Waye and Willard, 1986) linked to a wholly synthetic alphoid monomer based on a published consensus sequence (Choo et al., 1991). The natural monomer contains a CENP-B binding motif (CENP-B box; Masumoto et al., 1989). In the synthetic monomer, this was replaced with a 42 bp tetracycline operator (tetO), the binding site for *E. coli* tetracycline repressor (tetR) (Figure 1A).

HAC formation in HT1080 cells requires input naked alphoid DNA of at least 30 kb for functional CENP-A core assembly (Okamoto et al., 2007). The artificial alphoid^tetO^ dimer cloned by conventional means to 3.5 kb was further extended by rolling circle amplification using ϕ29 phage DNA polymerase plus yeast transformation-associated recombination (TAR) cloning (Figure 1B; Ebersole et al., 2005). This yielded 50 kb of alphoid^tetO^ dimeric repeat cloned head-to-tail in a BAC vector (BAC32-2-mer(tetO); see Figure S1 in the Supplemental Data available with this article online).

BAC32-2-mer(tetO) formed HACs when introduced into HT1080 cells. FISH analysis of the transformants with probes specific for alphoid^tetO^ dimer or BAC sequences revealed HACs in 2 of 46 transformant cell lines analyzed (Figure 1C and Table S1A). Both the HAC formation efficiency (4.3%) and fraction of HAC-containing cells (35.7% or 28.6%) in the two cell lines were lower than control values obtained using a natural alphoid DNA array (30% and > 50%, respectively, for a 60 kb BAC containing wild-type chromosome 21 type I alphoid DNA [alphoid^11-mer^]). The alphoid^tetO^ HACs contained 16 or 48 copies of the input DNA (Table S1B), and lacked host chromosomal DNA detectable with inter- and intra-*Alu* PCR probes.

Subcloning of these two original cell lines yielded several cell lines in which one copy of the HAC was maintained stably under nonselective conditions (loss rate = 0.0024 or 0.0054, Table S1B). Subsequent experiments were performed on alphoid^tetO^ HAC-containing cell line AB2.2.18.21. FISH analysis with the BAC probe revealed that the HAC behaved normally during mitosis (Figures 1D and 1E). Thus, despite the low HAC formation efficiency, the alphoid^tetO^ HAC segregates correctly as an independent chromosome.

### Tetracycline Repressor and Centromere Proteins Associate with Alphoid^tetO^ Sequences in the HAC Kinetochore

To test the ability of tetR to target the alphoid^tetO^ array within the active kinetochore, constructs expressing fluorescent proteins (FP) fused to tetR (mRFP-tetR and tetR-EYFP) were transfected into AB 2.2.18.21 cells. After expression for 24 hr, mRFP-tetR showed a single bright focus in interphase nuclei (Figures 1F′ and 1G′). This was the HAC, as it colocalized with both centromere and kinetochore markers, including CENP-B and CENP-C (Figures 1F and 1G). A specific signal could also be seen on the HAC in mitotic cells (Figure S2).

Immuno-FISH (Figure S3) confirmed that the HAC (detected with the BAC probe) binds kinetochore proteins, including CENP-A, CENP-C, and CENP-H (Warburton et al., 1997; Sugata et al., 2000), as well as INCENP, a key subunit of the chromosomal passenger complex (Vagnarelli and Earnshaw, 2004).

Together, these results confirm that the alphoid^tetO^ HAC assembles a bona fide kinetochore that can be targeted with tetR fusion proteins.

### Several Different Chromatin Structures Form on the Alphoid^tetO^ HAC

**Figure 2:**
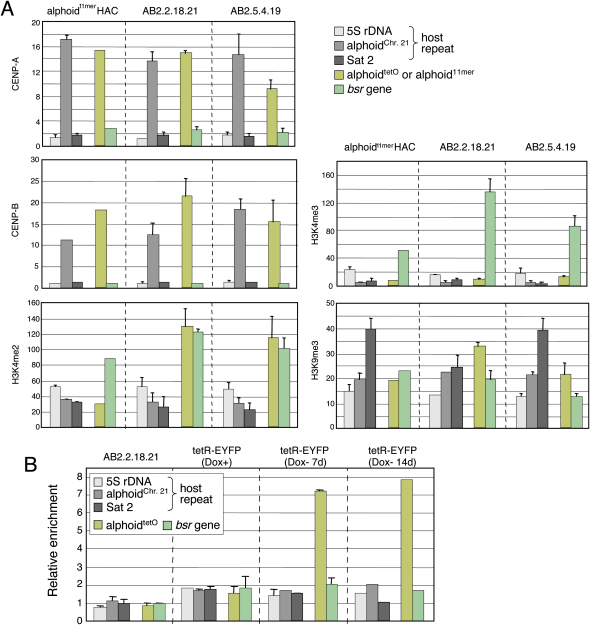
ChIP Analysis of CENP Assembly and Modified Histone H3 on Two Independent Alphoid^tetO^ HACs (A) PCR analysis of immunoprecipitates using antibodies against CENP-A, CENP-B, H3K4me2, H3K4me3, or H3K9me3. Chromatin was precipitated from three HAC-containing cell lines: a control HAC with a 60 kb BAC array of chromosome 21 type I 11-mer alphoid DNA (alphoid^11-mer^) (left), or the independent AB2.2.18.21 and AB2.5.4.19 sublines bearing the alphoid^tetO^ HAC (middle and right, respectively). The bars show the relative rate of recovery of various target DNA loci by immunoprecipitation with each antibody, calculated by dividing the percentage recovery of each DNA locus (as indicated) by that recovered in the control IP with mouse normal IgG. Error bars indicate SD (n = 3). CENP-A and CENP-B associate preferentially with the alphoid^tetO^ DNA, alphoid^11-mer^ from the wild-type control HAC, or endogenous alphoid^chr. 21^ relative to that seen with the *marker* genes, 5S ribosomal DNA and Sat2 (p < 0.05). The average recoveries of alphoid^tetO^ sequences by anti-H3K4me2 antibody were significantly higher than the recovery of the alphoid^11-mer^ from the control HAC and alphoid^chr. 21^ (p < 0.05, Student's t test). (B) AB2.2.18.21 cells bearing the alphoid^tetO^ HAC and expressing tetR-EYFP were cultured in doxycycline-free medium for 7 or 14 d and analyzed by ChIP assay with anti-GFP antibody. The bars show the relative enrichment of various target DNA loci by immunoprecipitation with anti-GFP antibody (binds the EYFP moiety of tetR-EYFP). Error bars indicate SD (n = 2).

Chromatin immunoprecipitation (ChIP) analysis confirmed that the synthetic alphoid^tetO^ monomers are located within the active kinetochore on the HAC. Immunoprecipitation with antibodies to CENP-A and CENP-B yielded an enrichment for the alphoid^tetO^ repeat comparable to that seen at control endogenous and HAC centromeres (Figure 2A). Analysis of extended chromosome fibers revealed that CENP-A is distributed along the length of the HAC, and is associated primarily with regions of alphoid^tetO^ sequence (Figure S4).

ChIP analysis revealed high levels of trimethylated histone H3 Lys4 (H3K4me3), a marker for transcriptionally active chromatin (Jenuwein and Allis, 2001; Santos-Rosa et al., 2002; Schneider et al., 2004), on the marker gene of the alphoid^tetO^ HAC. Much lower H3K4me3 levels were found on the alphoid^tetO^ repeat itself (Figure 2A). Consistent with this, trimethylated histone H3 Lys9 (H3K9me3), a marker for silent chromatin (Peters et al., 2003; Guenatri et al., 2004), was associated with the alphoid^tetO^ array (Figure 2A), where it alternated with regions containing CENP-A in extended HAC fibers (Figure S4). Similar results were obtained with the 11-mer alphoid repeat of native chromosome 21 (alphoid^chr. 21^) and the alphoid^11-mer^ repeat of the control HAC (Figure 2A).

H3K4me2, a marker for transcriptionally competent or neutral chromatin (Santos-Rosa et al., 2002; Schneider et al., 2004), has recently been proposed to be a marker for centromere chromatin (Sullivan and Karpen, 2004; Schueler and Sullivan, 2006). Indeed, regions containing H3K4me2 tended to alternate with regions containing CENP-A in extended HAC fibers (Figure S4). However, we found H3K4me2 associated with the alphoid^tetO^ repeat at levels significantly above those on alphoid^chr. 21^ or the alphoid^11-mer^ control HAC (Figure 2A). Thus, the alphoid^tetO^ array appears to form a slightly more open/neutral chromatin structure than occurs at native centromeres. However, because the alphoid^tetO^ and control HAC exhibit comparable high stability, these subtle differences must not interfere with kinetochore function.

Direct binding of tetR-EYFP to the alphoid^tetO^ array in AB2.2.18.21 cells stably expressing tetR-EYFP fusion protein was confirmed by ChIP analysis using anti-GFP antibody (Figure 2B). A stable subline was isolated and maintained in medium containing doxycycline (Dox) to inhibit tetR-EYFP binding to the alphoid^tetO^ array during selection and growth of the cells. Anti-GFP antibody precipitated alphoid^tetO^ DNA fragments only after the cells were grown without doxycycline for 7 and 14 d (Figure 2B). Thus, targeting of the tetR to the alphoid^tetO^ DNA in vivo can be controlled by the presence or absence of doxycycline.

### Binding of the tTA Transactivator Can Induce Alphoid^tetO^ HAC Loss

Targeting of the transcriptional transactivator tTA to the alphoid^tetO^ HAC produced a mosaic response in which the HAC kinetochore was inactivated in some cells, but not in others.

**Figure 3:**
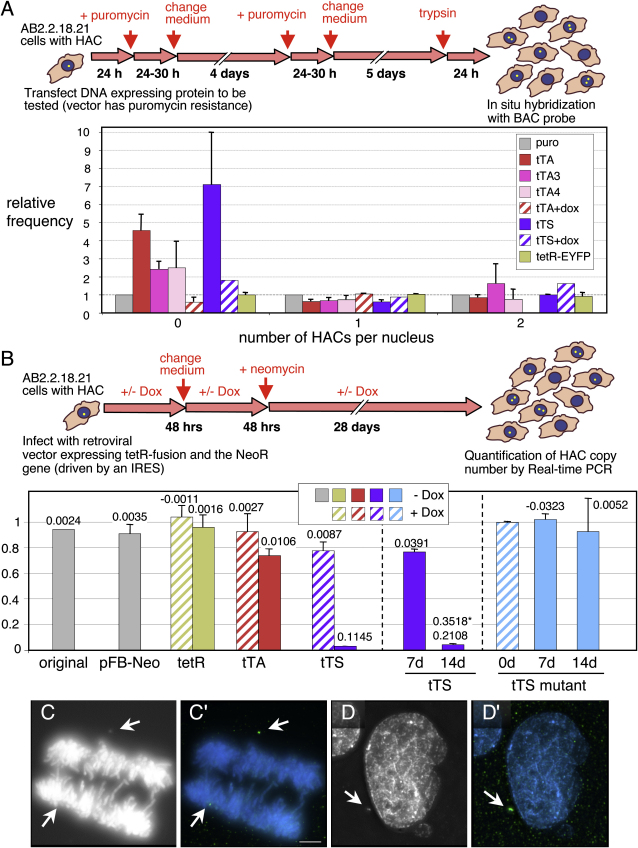
Targeting a Transcriptional Activator or Silencer into the HAC Kinetochore Induces HAC Loss (A) The HAC cell-by-cell stability assay. Coding regions of proteins to be tested were cloned into a vector that also expresses a puromycin resistance marker. Nontransfected cells were killed by puromycin and the remaining population was analyzed by FISH to quantify the HAC retention after 11–12 d of culture. To correct for variability in transfection and killing efficiency, all values were normalized to the results of transfection with empty vector bearing puromycin resistance (gray bars in lower panel). Lower panel: expression of transcriptional activators (tTA, tTA3, tTA4) and silencer tTS causes significant destabilization of the HAC with efficiency tTS > tTA > tTA3,tTA4 (n = 2–5). Constructs yielding results indistinguishable from the control have a value on the ordinate of 1.0. Addition of doxycycline, which prevents TetR from binding TetO, blocked the inactivation of HAC kinetochore by tTA and tTS (shaded bars). (B) Quantitative HAC stability assay using real-time PCR. The proteins to be tested were expressed using retroviral vectors. Virus-infected cells were maintained in medium containing neomycin ± doxycycline. After 30 d postinfection (left panel) (or additionally, 7 or 14 d for tTS or tTS mutant expressing cells, middle and right panels), the relative copy number of the alphoid^tetO^ array was quantitated by real-time PCR. Numbers above the bars indicate the HAC loss rate (R) calculated using the formula: N_30_ = N_0_ × (1−R)^30^. Asterisk indicates HAC loss rate between 7 and 14 d after tTS binding. Cells expressing the tetR protein (tetR) or infected with empty vector (pFB-Neo) showed no HAC instability after 30 d of culture. The KAP-1 interaction deficient mutant (tTS mutant, right blue bars) failed to induce HAC instability over 14 d. Error bars indicate SD (n = 3). (C and C′) The HAC (detected by FISH, green in [C′]) fails to segregate with the bulk chromosomes (stained with DAPI, grayscale in [C], blue in [C′]) in anaphase cells expressing the tTA transactivator. Size bar = 5 μm. (D and D′) Nanonucleus revealed by DAPI staining (D) contains the HAC (D′), as revealed by FISH with the BAC probe (colors as in [C′]). HACs are indicated by arrows.

In a “cell-by-cell” assay, we transfected cells bearing the alphoid^tetO^ HAC with various plasmids, killed the nontransfected cells with puromycin, and scored the percentage of cells bearing 0, 1, or 2 copies of the HAC (detected by FISH with the BAC probe; Figure 3A) after 11–12 d. To control for effects of the transfection procedure on HAC stability, all values were normalized to those for cells transfected with plasmid carrying only a puromycin resistance cassette.

Transfection of cells with plasmids expressing tetR-EYFP had essentially no effect on HAC stability (Figure 3A). In contrast, tTA expression caused a reproducible increase in the population of cells lacking the HAC (Figure 3A). Transfection with tTA3 (39% as active as tTA in promoting transcription) or tTA4 (14% activity) also caused significant, albeit reduced, levels of HAC loss. The tTA had no effect on HAC stability if cells were grown in the presence of doxycycline.

In a quantitative population assay, AB2.2.18.21 cells were infected with retroviral vectors expressing the tTA, and the copy number of alphoid^tetO^ dimer in selected clones was quantitated 30 d later by real-time PCR. Cells infected with control vectors expressing the marker gene or tetR alone showed no increase in HAC instability (Figure 3B). Cultures expressing the tTA showed a 6-fold increase in the rate of HAC loss (27% decrease in HAC content). No significant HAC loss was observed in parallel cultures grown in doxycycline (Figure 3B).

tTA expression caused a mosaic pattern of alphoid^tetO^ HAC loss in clonal cell lines after culturing for 37 d without selective drug (Figure S5). Chromosome loss rates were remarkably high in 43% (10/23) of cell lines, but were either moderate or normal in the remaining cell lines. In controls, expression of mRFP-tetR had a minimal effect on the stability of the alphoid^tetO^ HAC, and expression of the tTA or mRFP-tetR had no effect on loss rates for endogenous chromosomes X or 17, thereby confirming that chromosome loss induced by tTA expression was specific for the alphoid^tetO^ HAC.

**Figure 4:**
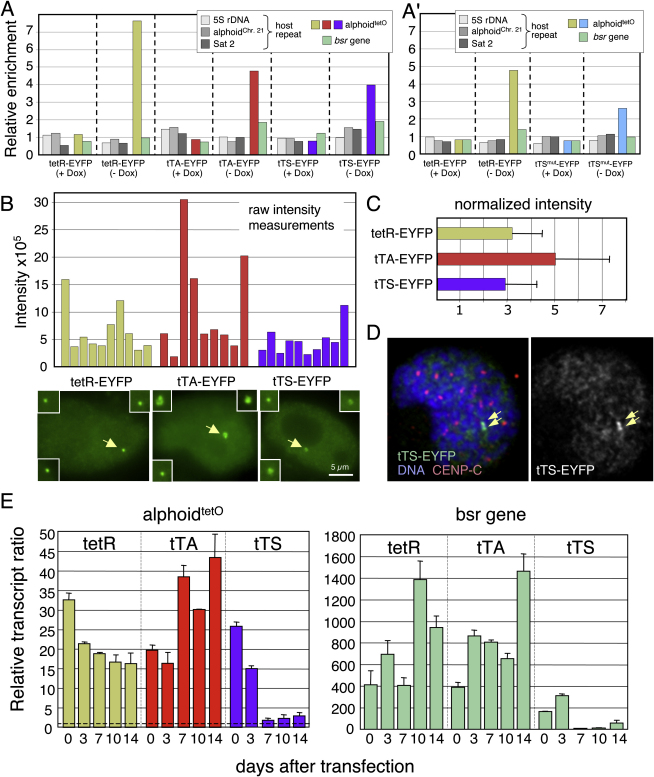
TetR-Fusion Proteins Specifically Associate with the Alphoid^tetO^ Array and Change Its Transcriptional Activity (A) AB2.5.30 cells containing a stably integrated alphoid^tetO^ dimer array and expressing tetR-EYFP, tTA-EYFP, or tTS-EYFP were cultured in doxycycline-free medium for 14 d and analyzed by ChIP using anti-GFP (binds EYFP). Bars show the relative enrichment of various target DNA loci. (A′) AB2.2.18.21 cells bearing the alphoid^tetO^ HAC and expressing tetR-EYFP and tTS mutant (tTS^mut^)-EYFP were cultured in doxycycline-free medium for 14 d and analyzed by ChIP with anti-GFP. The bars show the relative enrichment of various target DNA loci by ChIP. All tetR-fusion proteins specifically bound to the alphoid^tetO^ DNA in a doxycycline-dependent manner. (B and C) Quantitative analysis of the fluorescence intensity of various tetR-EYFP constructs bound to the HAC. Transfected cells were analyzed using a DeltaVision microscope under identical acquisition conditions. (B) The raw fluorescence intensity of the tetR-EYFP constructs is shown (n = 9–10), together with representative images. Size bar = 5 μm. (C) The fraction “enriched” on the HAC was calculated by normalizing for the unbound nucleoplasmic fraction. In some cells, the amount of tTA bound was relatively higher. In these cells, the HACs also appeared larger (insets in [B]), possibly because they contain more decondensed chromatin. (D) Mitotic cells with tTS-EYFP bound to the HAC. The presence of two sister chromatids confirms that the HAC had replicated in the previous S phase. Arrowheads indicate the HAC. (E) Levels of transcripts derived from the alphoid^tetO^ HAC were analyzed by quantitative RT-PCR using total RNA purified from AB2.2.18.21 cells expressing tetR, tTA, or tTS. Cells were cultured in doxycycline-free medium for 3, 7, 10, and 14 d. The bars show the transcription levels of alphoid^tetO^ dimer (left panel) or *bsr* gene (right panel) relative to that of the negative control, which contains no reverse transcriptase in the reaction mixture (dashed line on left panel). Levels of each transcript were corrected for the copy number of alphoid^tetO^ HAC as determined by quantitative PCR using the genomic DNA of each cell line and normalized by that of endogenous alphoid^chr.21^ (for alphoid^tetO^) or human *β-actin* (for *bsr* gene). The levels of transcripts from alphoid DNA were extremely low (∼2.9-fold above the reverse transcriptase free control reaction for alphoid^chr.21^) compared to those from the *bsr* gene (∼1,500 fold) and *β-actin* (∼19,000 fold). Error bars indicate SD (n = 2).

tTA expression caused a ∼2-fold increase in the very low level of alphoid^tetO^ transcripts, but had no effect on the much higher levels of transcription from the *bsr* gene (Figure 4E). However, examination of the HAC chromatin following tTA expression failed to reveal significant structural differences detected by ChIP with antibodies to H3K4me2 or H3K4me3 (data not shown). This is most likely because tTA binding to the HAC elicits a mosaic response, in which a minority of cells respond by increasing transcription, and destabilizing the HAC (Figures 4B and 4E and Figure S5B′). Since the HAC appears to resist effects caused by tTA binding in most cells, this could explain why the chromatin changes analyzed by ChIP remain below our limit of detection.

These experiments demonstrate that the alphoid^tetO^ HAC kinetochore can be inactivated in a subset of cells by targeting its chromatin with a transcriptional activator. If, indeed, kinetochore inactivation is induced by transcription of the alphoid^tetO^ array, this result might be similar to that observed in budding yeast, where strong transcriptional bombardment can inactivate a conditional centromere (Hill and Bloom, 1987). What was more surprising was that a transcriptional silencer had a much stronger effect and appeared to completely inactivate the kinetochore.

### Binding of the tTS Silencer Induces Dramatic Alphoid^tetO^ HAC Loss

The tTS, a powerful transcriptional repressor, induced a highly penetrant destabilization of the HAC (Figures 3A and 3B). The tTS is a fusion of the tetR and the Kruppel-associated box (KRAB)-AB silencing domain of the Kid-1 protein (kidney, ischemia, development; Witzgall et al., 1994; Freundlieb et al., 1999).

Unlike the variable and weak effects seen following expression of the tTA, AB2.2.18.21 cells showed a 72-fold increase in the rate of HAC loss (97% decrease in HAC content) 30 d after infection with retroviral vectors expressing the tTS (Figure 3B). HAC loss was essentially complete by 14 d of tTS expression. This effect of the tTS was also seen in the “cell-by-cell” protocol of Figure 3A. Expression of a tTS point mutant (tTS^mut^) unable to bind the corepressor KAP-1 (KRAB-associated protein-1; Agata et al., 1999; Matsuda et al., 2001) had no effect on alphoid^tetO^ HAC stability even after 14 d (Figure 3B). Thus, destabilization of the HAC by the tTS apparently occurs via the KAP-1 pathway.

In control experiments, we determined that the differential effects of tetR-EYFP, tTA, and tTS on HAC stability could not simply be explained by differences in the affinity of these proteins for the alphoid^tetO^ array. In ChIP experiments, tetR-EYFP appeared to bind to the alphoid^tetO^ array slightly more efficiently than did tTA-EYFP and tTS-EYFP (Figure 4A). The three proteins exhibited similar levels of binding to the HAC in vivo as measured by quantitative fluorescence (Figures 4B and 4C). However, in some cells, HACs with bound tTA-EYFP appeared to be larger, and had higher levels of associated FP. Importantly, the tTS, which dramatically destabilized the HAC, bound to it at levels comparable to the tetR, which had no observable effect on HAC stability.

### The tTS Disrupts CENP-A Kinetochore Chromatin

**Figure 5:**
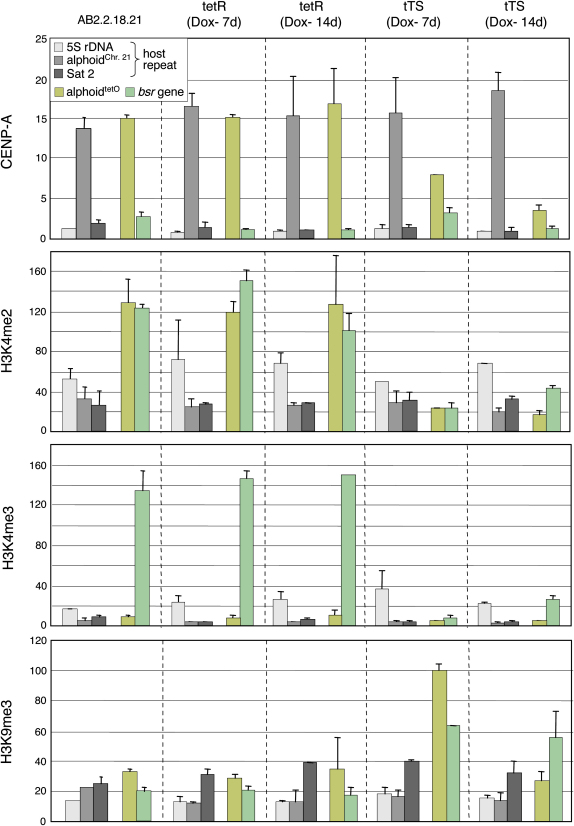
tTS-Enhanced Heterochromatin Assembly and CENP-A Chromatin Disassembly on the Alphoid^tetO^ HAC AB2.2.18.21 cells expressing tetR or tTS were cultured in doxycycline-free medium for 7 or 14 d and analyzed by ChIP with antibodies against CENP-A and modified histone H3. CENP-A assembly on alphoid^tetO^ dimer was gradually decreased following induction of tTS binding for 7–14 d. Levels of H3K4me2 and H3K4me3 on the alphoid^tetO^ HAC decreased rapidly upon tTS binding. In contrast, H3K9me3 levels on the alphoid^tetO^ HAC increased transiently following tTS binding, but then declined again (possibly due to loss of the HAC). Error bars indicate SD (n = 2–3).

tTS binding to the alphoid^tetO^ array converted the centromeric chromatin to a more heterochromatic state, disrupting the CENP-A kinetochore domain (Figures 4 and 5). This conversion was accompanied by dramatic reduction of levels of alphoid^tetO^ transcription detected by quantitative RT-PCR (Figure 4E). tTS binding caused a rapid loss of H3K4me2 and somewhat slower decrease in CENP-A levels on the alphoid^tetO^ array (Figure 5). We also observed a transient increase in H3K9me3 levels. In controls, binding of tetR to the alphoid^tetO^ HAC caused no detectable changes in the HAC chromatin.

tTS binding also reduced H3K4me2 and H3K4me3 levels and increased H3K9me3 levels on the marker gene as well as strongly repressing its transcription (Figures 4 and 5). Thus, the closed chromatin structure induced by tTS binding spreads laterally to the BAC vector region. Furthermore, although only 4% of the HAC DNA remained in the population at later times, the fact that H3K9me3 levels remained high on the marker gene while dropping on alphoid^tetO^ DNA suggests that even after tTS binding, subtle differences in chromatin structure remain between the (now inactive) kinetochore and flanking regions.

### Kinetochore Inactivation Leads to HAC Loss by Nondisjunction and Formation of Nanonuclei

**Figure 6:**
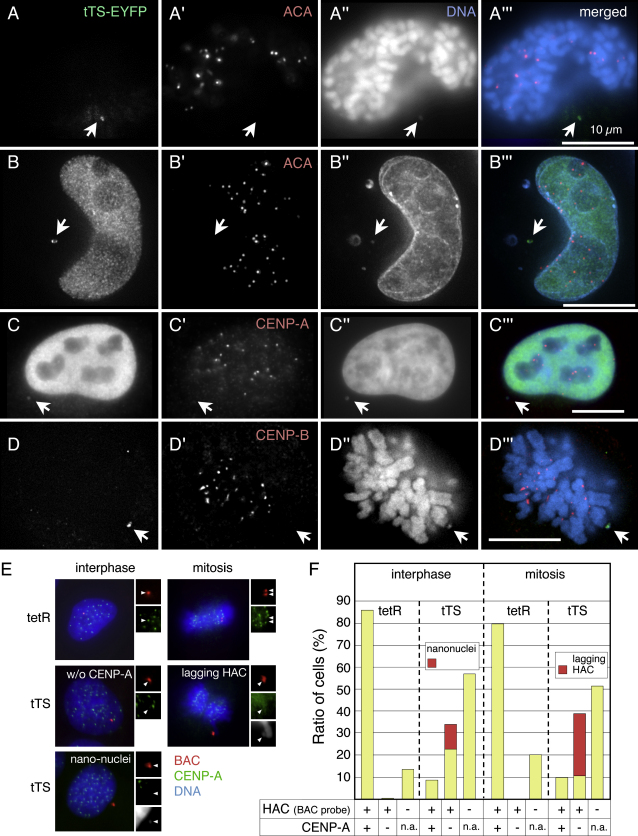
tTS Targeting to the Alphoid^tetO^ HAC Induces Lagging Chromosomes and Formation of Nanonuclei AB2.2.18.21 cells were either transiently transfected with constructs expressing the tTS ([A], [B], and [D]) or AB2.2.18.21 cells stably expressing the tTS (C) were incubated in the absence of doxycycline to allow TetR binding to the tetO sequences in the alphoid^tetO^ HAC. EYFP (green) was detected either via its own fluorescence ([A], [B], and [D]) or using an anti-GFP antibody (C). Cells were also stained for CENP antigens with ACA (red in [A′ and B′]), anti-CENP-A antibody (red, [C′]), or anti-CENP-B antibody (red, [D′]). Chromosomes were stained with DAPI (blue, [A″–D″]). Nonaligned alphoid^tetO^ HACs targeted by tTS-EYFP were observed in mitotic cells (A and D). These HACs lacked detectible CENP-A, -B, and -C. In interphase cells, nanonuclei with associated tTS-EYFP were observed ([B], [C], and [E]). Nanonuclei also lacked detectible CENP-A and ACA antigens. Arrows indicate the HACs. Bar = 10 μm. (E) AB2.2.18.21 cells with alphoid^tetO^ HAC expressing tetR (upper panels) or tTS (middle and lower panels) were cultured in doxycycline-free medium for 7 d and analyzed by immuno-FISH using anti-CENP-A antibody (green) and BAC vector probe (red). DNA was stained by DAPI (blue). Arrowheads indicate the alphoid^tetO^ HAC. In tTS expressing cells, lagging HACs (middle right) and nanonuclei (lower left) were observed in mitosis and interphase, respectively. (F) Nanonuclei or lagging HAC were frequently observed in tTS-expressing cells (red bars, n > 50).

**Figure 7:**
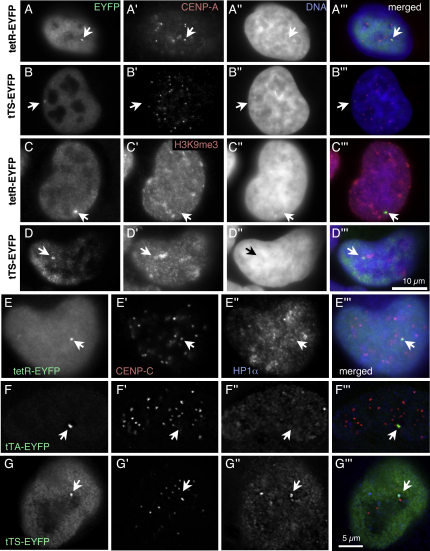
tTS Binding Induces Heterochromatin Formation and Kinetochore Disassembly on the Alphoid^tetO^ HAC (A–D) Alphoid^tetO^ HAC cell lines expressing tetR-EYFP (A and C) or tTS-EYFP (B and D) were cultured in doxycycline-free medium for 8 d and analyzed by indirect immunofluorescence using anti-GFP (green, [A–D]) and anti-CENP-A (red, [A′ and B′]) or anti-H3K9me3 (red, [C′ and D′]). DNA was stained by DAPI (blue, [A″–D″]). The HAC is indicated by arrows. Bar in (D″′) = 10 μm for (A–D). (E) tetR-EYFP has no effect on kinetochore structure and does not recruit HP1α. (F) tTA-EYFP inactivates the kinetochore, as shown by loss of CENP-C, but does not recruit HP1α. (G) The tTS inactivates the kinetochore, accompanied by a robust recruitment of HP1α. Bar in (G″′) = 5 μm for (E–G).

The tTS induces HAC loss by disrupting kinetochore structure. If AB2.2.18.21 cells expressing tTS-EYFP were cultured in doxycycline-free medium for 7–8 d to allow tTS binding to the kinetochore, the CENP-A, CENP-B, and CENP-C signals on HACs with associated FP signals were either undetectable or significantly weaker than those on endogenous centromeres (Figures 6, 7B, and 7G and Figure S6C). Alphoid^tetO^ HACs lacking detectable CENP-A were observed in 34% of interphase cells and 39% of mitotic cells (Figures 6E and 6F). In those cells, the FP signal overlapped with significant levels of H3K9me3, a marker for heterochromatin that was not enriched on HACs targeted by tetR-EYFP (Figures 7C and 7D). In parallel control experiments, tetR-EYFP colocalized with CENP-A and CENP-C on the alphoid^tetO^ HAC, confirming that this protein does not disrupt kinetochore structure (Figures 6E and 7A, and Figures S6A and S6B).

In cells expressing the tTA, we frequently observed that one or both copies of the HAC was spatially separate from the other chromosomes during mitosis (Figure 3C). Similarly, when AB2.2.18.21 cells were examined after 7–8 d of tTS-EYFP expression, we saw HACs with FP signals lying outside the mass of congressed chromosomes in 28% of mitotic cells (Figures 6A, 6D, and 6F). These nonaligned HACs lacked detectible kinetochore proteins, including CENP-A, CENP-B, and CENP-C.

Loss of the alphoid^tetO^ HAC induced by the various forms of tTA and tTS could occur by two mechanisms: nondisjunction (2:0 segregation) or chromosome loss (failure to replicate , 1:0 segregation). All of our assays revealed that the increase in cells with no HAC was not balanced by a corresponding increase in cells with two HACs. (Note that the cell-by-cell assay scores only HAC signals in the nucleus. See below.) Thus, the HAC initially appeared to be undergoing chromosome loss. However, the fact that tetR-EYFP had no effect on HAC stability suggested strongly that tetR binding to the alphoid^tetO^ array does not interfere with HAC replication, and that loss must be occurring via another mechanism. This is consistent with data shown in Figure 4D in which a HAC with bound tTS is present as two replicated sister chromatids in mitosis.

Detailed examination of cultures revealed that cells expressing either the tTA or tTS often had minute DAPI-stained structures in the cytoplasm (Figures 3D, 6B, 6C, and 6E and Figure S6E). These resemble tiny versions of micronuclei typically observed when chromosomes fail to segregate correctly in mitosis, and we refer to them as nanonuclei. After 7 d of tTS expression, 11% of interphase cells had nanonuclei containing the alphoid^tetO^ HAC (Figures 6B, 6C, 6E, and 6F and Figure S6E). Nanonuclei lack detectible ACA, CENP-A, or CENP-B staining, indicating that they do not contain functional kinetochores. Although detailed characterization of nanonuclei is beyond the scope of this study, we suspect that sequestration of the alphoid^tetO^ HAC in these tiny structures ultimately results in its loss from the population.

Together these results indicate that targeting of chromatin-modifying activities into the alphoid^tetO^ kinetochore disrupts centromere chromatin, evicting CENP-A, CENP-B, and other kinetochore proteins, and ultimately inactivating the kinetochore.

### HP1α Recruited by the tTS Can Inactivate the Kinetochore

**Figure 8:**
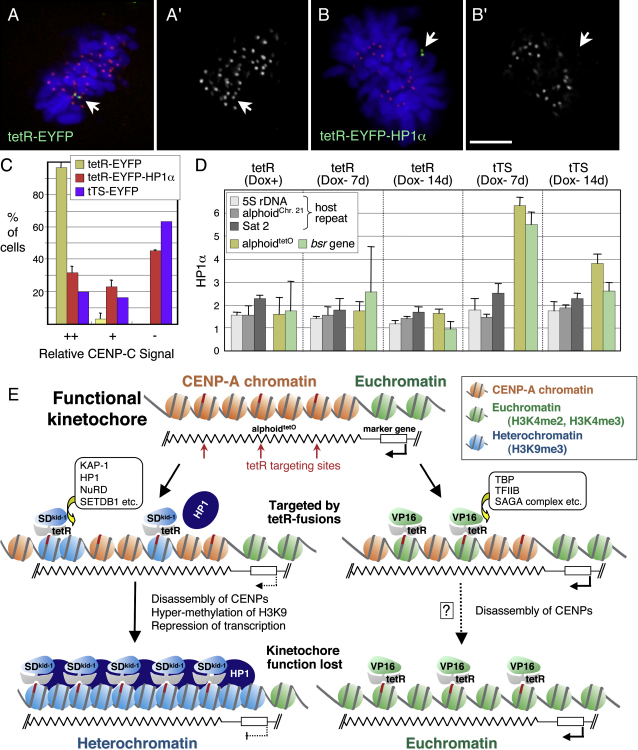
HP1α Recruitment Inactivates the Alphoid^tetO^ Kinetochore (A) A duplicated HAC at metaphase has bound tetR-EYFP and CENP-C. (B) A duplicated HAC at metaphase has bound tetR-EYFP-HP1α, but lacks CENP-C. Bar in (B′) = 5 μm for (A and B). (C) Quantitation of the effects of expression of tetR-EYFP, tetR-EYFP-HP1α, and tTS-EYFP on the levels of CENP-C at kinetochores. (D) AB2.2.18.21 cells expressing tetR or tTS were cultured in doxycycline-free medium for 7 or 14 d and analyzed by ChIP with anti-HP1α antibody. The tTS induces targeting of HP1α to both the alphoid^tetO^ array and the adjacent marker gene. The decrease seen at 14 d likely reflects loss of the HAC. Error bars indicate SD (n = 2–3). (E) Inactivation of the kinetochore by modulating the epigenetic status of the underlying chromatin. Centromere chromatin containing CENP-A (red) and H3K4me2 (not shown here) assembles on the alphoid^tetO^ dimer array of the HAC, forming an active kinetochore structure. The chromatin of the marker gene contains H3K4me3 (green). Binding of the tTS (tetR-SD^kid-1^) induces H3K9 trimethylation (blue) at its target sites (red rectangle and arrow) on the alphoid^tetO^ HAC. The resulting remodeling and compaction of the chromatin is incompatible with the structure of CENP-A chromatin and CENP-A quickly disappears from the heterochromatic alphoid^tetO^ array. The centromere/kinetochore is inactivated. tTA (tetR-VP16) binding induces formation of open chromatin at the target site. In some cases, the open chromatin structure (euchromatin) somehow disrupts the CENP-A chromatin. However, in the case of tTA, the degree of HAC inactivation was less than that seen with the tTS. This suggests that the core of CENP-A chromatin is less sensitive to chromatin opening induced by VP16 and that in many cases HACs with open chromatin can still maintain a functional kinetochore. The data presented suggest an epigenetic mechanism to regulate the kinetochore activity based in part upon antagonism between centromere chromatin rich in CENP-A and H3K4me2 and heterochromatin rich in H3K9me3.

tTS binding to the alphoid^tetO^ array could in theory inactivate the kinetochore by a number of pathways, including the formation of heterochromatin involving H3K9me3 and HP1α. We therefore stained HAC-bearing cells for HP1α following exposure to tetR, the tTA, and the tTS (Figure 7). Of these treatments, only the tTS induced a robust accumulation of HP1α at the kinetochore (Figures 7G″ and 8D). This accumulation was accompanied by disruption of the kinetochore, as detected by the loss of CENP-C staining.

To test whether the accumulation of HP1α was sufficient to disrupt kinetochore structure, we fused tetR-EYFP to HP1α, thereby targeting the heterochromatin protein by a mechanism independent of core histone modifications. Strikingly, this directed targeting of tetR-EYFP-HP1α to the alphoid^tetO^ repeats was sufficient to disrupt kinetochore structure, as determined by the loss of CENP-C staining (Figure 8B). When cells were scored for the loss of CENP-C staining, the tTS and HP1 were found to yield a comparable effect (Figure 8C). Targeting of HP1α to the alphoid^tetO^ array did not block replication of the HAC, as detected by the presence of two paired sister chromatids (Figure 8B), but did destabilize the HAC (S.C., Jan Bergmann, M.N., Marcella Cervantes, V.L., H.M., and W.C.E., unpublished data). All these results suggest that HP1α recruitment may be sufficient to account for the action of the tTS and that the nucleation of heterochromatin within the array is sufficient to inactivate the kinetochore.

## Discussion

We have developed a novel human artificial chromosome (HAC) to manipulate for the first time, to our knowledge, the epigenetic status of a single human kinetochore. The HAC was based on a synthetic repeated DNA sequence, the alphoid^tetO^ dimer. One part of this dimer is a natural human alphoid monomer from chromosome 17 containing a CENP-B box. The other has no known counterpart in the natural human genome, and contains a 42 bp tetracycline operator (tetO) in place of the CENP-B box.

The kinetochore of the alphoid^tetO^ HAC is functionally indistinguishable from the kinetochore of endogenous chromosomes. It binds several key markers for centromeric chromatin, including CENP-A, CENP-C, and CENP-H, as well as H3K4me2. It apparently also contains some normal heterochromatin associated with H3K9me3 and INCENP. Most importantly, the alphoid^tetO^ HAC exhibits normal behavior in mitosis and normal stability in long-term culture.

The tetO sequences within the active kinetochore of the alphoid^tetO^ HAC remain accessible to tetracycline repressor (tetR). Furthermore, targeting of mRFP or EYFP into the kinetochore as tetR fusions had at most a minimal effect on HAC stability. However, targeting of the tTA transcriptional activator or tTS transcriptional repressor inactivated the HAC kinetochore. Thus, this HAC is, to our knowledge, the first chromosome outside of budding yeast (Hill and Bloom, 1987) with a conditional centromere.

### Inactivation of the Kinetochore by a Transcriptional Activator

Targeting of the tTA to the alphoid^tetO^ array inactivates the kinetochore in some cells. The VP16 transactivation domain of the tTA can directly interact with several general transcription factors including the TATA-binding protein (TBP), TFIIB, and the SAGA histone acetylase complex in vivo (Hall and Struhl, 2002; Klein et al., 2003; Herrera and Triezenberg, 2004). tTA inactivation of the kinetochore could involve either transcriptional activation, or simply induction of a transcriptionally competent open chromatin structure around its binding site (Figure 8E).

The tTA appeared to destabilize the HAC in some cells but not in others, resulting in a less potent effect overall on HAC stability than the tTS. We have considered two explanations for this. First, CENP-A chromatin may be able to resist transcriptional activation by the tTA. Alternatively, CENP-A chromatin may tolerate a degree of chromatin “opening” while retaining kinetochore activity. The presence of actively transcribed genes within rice centromeres (Nagaki et al., 2004) and human neocentromeres (Saffery et al., 2003), plus the detection of low level transcription of alphoid sequences in humans (Figure 4E) or in DT40 cells with a human chromosome (Fukagawa et al., 2004), suggest that in many cases, functional kinetochores can contain “open” chromatin. Possibly, low levels of transcription are tolerated within kinetochore chromatin, however high level transcription triggered by a stochastic event occurring in a subset of cells expressing the tTA may disrupt kinetochore function. It has long been known that bombardment with transcription from a strong promoter can inactivate a yeast kinetochore (Hill and Bloom, 1987).

### Heterochromatin Is Incompatible with Kinetochore Function

The tTS was a significantly stronger disruptor of kinetochore function than the tTA (Figure 8E). The tTS can recruit the scaffolding protein KAP-1 (Friedman et al., 1996), which in turn recruits chromatin modification factors including Mi-2α— a core component of the NuRD histone deacetylase complex (Schultz et al., 2001), SETDB1—the histone H3 Lys9 selective methyltransferase (Schultz et al., 2002), and heterochromatin protein 1 isoforms (Nielsen et al., 1999; Ryan et al., 1999; Lechner et al., 2000). In controls, a tTS point mutant unable to interact with KAP-1 did not destabilize the alphoid^tetO^ HAC, suggesting that recruitment of KAP-1 can lead to inactivation of the alphoid^tetO^ kinetochore. We will report elsewhere a detailed analysis of the role of the various KAP-1 domains in kinetochore inactivation (S.C., Jan Bergmann, M.N., Marcella Cervantes, V.L., H.M., and W.C.E., unpublished data).

tTS disruption of the kinetochore is unlikely to be a passive byproduct of the recruitment of bulky chromatin remodeling complexes onto the alphoid^tetO^ array. We have shown that a variety of proteins, including tTS^mut^ can be recruited into the alphoid^tetO^ kinetochore without having any detectable effect on its function. Furthermore, it is important to note that the tTA also recruits a different spectrum of chromatin remodeling complexes into the kinetochore, yet has a much milder effect on kinetochore function.

It appears that the tTS inactivates the alphoid^tetO^ kinetochore by nucleating the formation of classical heterochromatin containing H3K9me3 and HP1. In a previous study, binding of a KAP-1 fusion protein to a targeted transgene reduced levels of histone H3K9 acetylation and H3K4 methylation, and increased H3K9me3 levels at the site (Sripathy et al., 2006). Here, we found that tTS binding decreased H3K4 methylation, increased H3K9me3 levels, and induced HP1 accumulation within the alphoid^tetO^ kinetochore. This heterochromatin-like state spread onto the flanking marker gene, which had previously been in an open, actively transcribing state. In fact, we found that targeting of HP1 directly as a fusion to tetR-EYFP was sufficient to inactivate the kinetochore. This rules out the necessity of other activities of the tTS or KAP-1 in kinetochore inactivation.

Although it was long taken for granted that centromeres were heterochromatic, recent cytological observations have suggested that this might not be the case (Schueler and Sullivan, 2006). In addition, it has been observed that overexpressed CENP-A does not incorporate into heterochromatin (Vermaak et al., 2002), and in *Drosophila*, heterochromatin can block neocentromere formation (Maggert and Karpen, 2001). The experiments shown here demonstrate for the first time, to our knowledge, that targeted nucleation of heterochromatin within the kinetochore abolishes the binding of the centromere specific histone H3, CENP-A, and inactivates a fully functional kinetochore. Although the detailed mechanism remains to be determined, we have considered three ways in which heterochromatin formation could evict CENP-A from the alphoid^tetO^ array. First, the conformation of chromatin fibers containing HP1 might be inconsistent with the relatively rigid structure proposed for CENP-A-containing chromatin (Black et al., 2004). In other studies, we have found that formation of H3K9me3 chromatin prevented CENP-A assembly on an ectopically integrated cluster of short alphoid DNA arrays (Okamoto et al., 2007), provided that CENP-B is present (Okada et al., 2007).

Alternatively, tTS binding leads to a rapid drop in the levels on the alphoid^tetO^ array of H3K4me2, which has recently been described as a marker for centromere chromatin (Sullivan and Karpen, 2004). This drop, which is balanced by a temporary increase of H3K9me3 levels, appears to slightly precede the loss of CENP-A from the alphoid^tetO^ array. This raises the possibility that H3K4me2, possibly together with other core histone modifications, could have an essential role in helping to maintain the structure of centromere chromatin.

Finally, heterochromatin assembly might inactivate the alphoid^tetO^ kinetochore by suppressing the (low) level of endogenous transcription of the array. Although evidence for RNAi involvement in vertebrate chromosome segregation is scant (Fukagawa et al., 2004; Kanellopoulou et al., 2005), RNAi has a clear role in establishment of the flanking heterochromatin at *S. pombe* kinetochores (Grewal and Jia, 2007).

Until now, the chromatin configuration of active kinetochores has been the subject of careful observation and intense speculation (Sullivan and Karpen, 2004), but has remained inaccessible to direct targeted manipulation. In addition to providing a clear demonstration that experimentally induced heterochromatin within the centromere is incompatible with kinetochore activity, our studies reveal that the remarkable dynamism of the chromatin within the kinetochore of the alphoid^tetO^ HAC should allow the systematic manipulation of the “histone code” within the kinetochore and definition of the full epigenetic signature of centromere chromatin.

## Experimental Procedures

Standard procedures, including descriptions of cell lines, the BAC transfection protocol, analysis of de novo HAC formation analyses by fluorescence in situ hybridization (FISH), construction of tetR-fusion proteins expression vectors, Chromatin immunoprecipitation (ChIP), real-time PCR, and indirect immunofluorescence are discussed in the Supplemental Experimental Procedures.

### Construction of tetO Dimer Alphoid BACs

The alphoid^tetO^ monomer sequence was assembled by coligation of five double-stranded oligomers and cloned into pBluescript using the *XhoI*/*SalI* sites. A naturally occurring alphoid monomer (168 bp) containing a consensus CENP-B box was isolated from p17H8 (Waye and Willard, 1986). The 2.7 kb higher-order repeat was digested with *BsmI* and the appropriate DNA fragment was isolated after agarose gel electrophoresis. The fragment was blunt-ended with T4 polymerase and cloned into the SmaI site of pBluescript. After amplification of the monomer by PCR to introduce SalI and *XhoI* sites, it was inserted into pBluescript next to the tetO monomer.

Subsequent cycles of digestion and cloning the *XhoI*/*SalI* site of pBluescript yielded p3.5α, containing 10 alphoid^tetO^ dimer fragments (Figure 1A). The extension of the tetO dimer alphoid repeat was carried out by rolling-circle amplification (RCA) for the circularized template of the alphoid^tetO^ 10-mer excised from p3.5α, using ϕ29 DNA polymerase. Resulting RCA products were cloned into a targeting vector in yeast by transformation-associated homologous recombination (TAR) according to previously described methods (Ebersole et al., 2005). The targeting vector (RSA/SAT43) contains YAC (*HIS3*, *CEN6*, *ARSH4*) and BAC (*Cm*, *ori F*) cassettes as well as a mammalian selectable marker (*bsr*).

Purified genomic DNA from the yeast clones was electroporated into *Escherichia coli* cells (DH10B, Invitrogen). The insert size of the synthetic alphoid^tetO^ DNA array was analyzed by PFGE (CHEF, Bio-Rad) after *NotI* digestion of BAC DNA.

### The Analysis of HAC Loss Rate, R, in the TetR-Fusion Protein Expressing Cells with Real-Time PCR

AB2.2.18.21 cells expressing tetR, tTA, and tTS fusion proteins were constructed by retroviral vector gene expressing system. Infected cells were treated by geneticin and/or 1 μg/ml of doxycycline for 30 d (and 7 or 14 d for tTS expressing cell). The genomic DNAs of each cell lines were purified using DNeasy Tissue Kit (QIAGEN) according to the manufacturer's instructions. Purified genomic DNAs were sonicated (Bioruptor sonicator, Cosmo Bio, Japan) for 3 min. One hundred nanograms of genomic DNA was used for analysis by real-time PCR with primers tet-1 and tet-3 using the iCyclerIQ™ MultiColor Real Time PCR Systems (Bio-Rad). The following primer sets were used: 5SDNA-F1 and 5SDNA-R1 for 5S ribosomal DNA, and tet-1 and tet-3 for the alphoid^tetO^ dimer.

### Analysis by FISH of the HAC Loss Rate, R, in Cells Expressing Cells TetR-Fusion Proteins

Cells were transfected with Lipofectamine 2000 (Invitrogen) at 90% confluence and after 24 hr puromycin was added to the medium at a concentration of 1.5 μg/ml. After 24–30 hr the medium was changed and the cells incubated for 4 d before being selected again with the same concentration of Puromycin for 24–30 hr. After the second selection, cells were incubated in fresh medium for 5 d, plated on poly-lysine-coated slides and processed for fluorescence in situ hybridization (FISH) using a BAC-based probe. The slides, fixed with Carnoy's fixative, were left to age overnight. The probe was denatured for 5 min at 95°C and added to the slides, which were incubated at 72°C for 2 min before overnight incubation at 39°C. After washes with 0.1 × SSC (20 × SSC: 3 M NaCl, 0.3 M NaCitrate) at 65°C followed by a wash with 4 × SSC + 0.1% Tween20 at RT, slides were incubated (with intervening washes), successively, with FITC-avidin, biotinylated-anti-avidin, and FITC-avidin. Slides were mounted with VectaShield.

## Supplemental Data

Document S1. Seven Figures, Two Tables, Supplemental Experimental Procedures, and Supplemental References
