# Supplementary material for: Inactivation of a Human Kinetochore by Specific Targeting of Chromatin Modifiers
Source: Dev Cell. 2008 Apr 15;14(4):507–22. doi: 10.1016/j.devcel.2008.02.001 (PMC2311382; doi:10.1016/j.devcel.2008.02.001)
Supplement: Document S1. Seven Figures, Two Tables, Supplemental Experimental Procedures, and Supplemental References [file mmc1.pdf]

Developmental Cell ***14***

Supplemental Data

Inactivation of a Human Kinetochore by Specific Targeting of Chromatin Modifiers

Megumi Nakano, Stefano Cardinale, Vladimir N. Noskov, Reto Gassmann, Paola Vagnarelli, Stefanie Kandels-Lewis, Vladimir Larionov, William C. Earnshaw, and Hiroshi Masumoto

Supplemental Experimental Procedures

Cell Lines

HT1080 cells (ATCC CCL121) were cultured in Dulbecco's Modified Eagle's Medium (DMEM, Invitrogen Corporation) supplemented with 10% (v/v) tet system-approved fetal bovine serum (Clontech Laboratories, Inc.) at 37°C in 5% CO_2. To construct cell lines expressing tetR-fusion proteins, mRFP-fused tetR protein expression vector, pRFP-tetR or tetR-fused VP16 transcriptional activation domain expression vector, ptTA was introduced into HT1080 sub-lines containing the alphoid^{tetO} HAC using the Polyfect Transfection Reagent (QIAGEN) according to the manufacturer's instructions. After transfection, cells were exposed to 4 μg/ml of puromycin for 2 days. A retroviral vector system was also used to obtain cell lines expressing tetR-fusion proteins. Infectious virus particles incorporating vectors expressing tetR-fusion proteins were generated according to the manufacturer's instructions and used to infect the AB2.2.18.21 or AB2.5.30 cell line. After infection, cells were treated with 150 μg/ml or 400 μg/ml of geneticin, respectively. The control HAC cell line W0210R-8 containing one copy of a stable HAC derived from synthetic wild-type 11-mer alphoid (alphoid^{11mer}) BAC was described previously.

BAC Transfection

The alphoid^{tetO} BAC DNAs were purified using a Qiagen large construction kit (QIAGEN). Using 4.5 μl of Lipofectamine Reagent (Invitrogen Corporation), 0.4 μg of purified BAC DNA was transfected into HT1080 cells. Bs-resistant cell lines were selected with 4 μg/ml blasticidin S hydrochloride (MP Biomedicals, Inc.) and analyzed by FISH. To obtain homogeneous populations of sub-lines containing HACs, single colonies were picked up from the original alphoid^{tetO} HAC clones AB 2.2.18 and AB 2.5.4. Throughout the sub-cloning process, cells were cultured in non-selective medium. Loss rates (R) of HACs per generation were calculated using the following formula: $N_{37}= N_0 \times (1\text{-}R)^{37}$. N_{37} is the average number of HACs per cell from 20 observed metaphase cells at day 37 and N_0=1 in this case because of the sub-cloning.

De novo HAC Formation Analyses by Fluorescence In Situ Hybridization (FISH)

Standard FISH techniques were carried out for the alphoid^{tetO} BAC-transformed cell lines as previously described (Masumoto et al., 1989). The probes used were PCR products of p3.5α for the alphoid^{tetO} dimer and RSA/SAT43 for the BAC vector DNA. Alphoid^{tetO} dimer template was amplified by PCR using TaKaRa LA Taq (Takara Bio Inc.) with M13 universal and reverse primers. Alphoid DNA hooks were eliminated from the BAC vector by restriction enzyme treatments and the fragment containing the YAC and BAC cassettes was purified from the gel. PCR amplified alphoid^{tetO} and the BAC vector DNA fragment were labeled using a nick-translation kit with digoxigenin-11dUTP or biotin16-dUTP (Roche Diagnostics). Images were

captured using a cooled-charge-coupled device (CCD) camera (Cool SNAP HQ, Photometrics) and analyzed by IPLab software (Signal Analytics).

Construction of tetR-Fusion Protein Expression Vectors

The tetR coding sequence of *E. coli* Tn10 was cloned with and without the stop codon into pZeoSV(-) (Invitrogen) using *EcoRI* and *BamHI*.

For cloning of retroviral expression vectors, the tetR coding sequence was amplified by PCR with Phusion High-Fidelity DNA polymerase (New England BioLabs) from pTet-tTS (Clontech) using primers with nuclear localization signal (NLS: PKKKRK), re-tetR-F (5'-ACGAATTCATGTCTAGATTAGATAAAAGTA-3') and re-tetR-R (5'-GAGGATCCCTAAGGCCTCTTTCTCTTCTTTTTTGGTTTA). The amplified tetR gene was inserted into the retroviral vector, pFB-Neo (Stratagene) using *EcoRI* and *BamHI* (pFB-tetR-Neo). The VP16 coding sequence was amplified from pTet-ON (Clontech), with PCR primers VP16-S (5'-GAAAGGCCTCTTAAATGTGAAAGTGGGTCC-3') and VP16-N (5'-GATCGCGGCCGCTCTACCCACCGTACTCGTCAATTC-3') and inserted into the pFB-tetR-Neo using *StuI* and *NotI* (pFB-tTA-Neo). The tTS coding sequence was amplified by PCR from pTet-tTS with primers, re-tetR-F and re-tTS-R (5'-CGCTCGAGCTAAGGCCTCCAGGGATCCTCTCCTTGCTGC-3') and inserted into the pFB-Neo using *EcoRI* and *XhoI* (pFB-tTS-Neo). The EYFP coding sequence was amplified by PCR from pEYFP-C1 (Clontech) and inserted into the pFB-tetR-Neo and pFB-tTS-Neo using *StuI* and *XhoI* (pFB-tetR-EYFP-Neo and pFB-tTS-EYFP-Neo). or inserted into the pFB-tTA-Neo using *NotI* (pFB-tTA-EYFP-Neo). tTS^{mut} has two amino acid mutation in KRAB-AB domain consensus sequence in SD^{kid-1} of tTS (Agata et al., 1999;

Matsuda et al., 2001). The pFB-tTS^{mut}-Neo and pFB-tTS^{mut}-EYFP-Neo were constructed by PCR using Phsion Site-Directed Mutagenesis Kit (NEB) with PCR primers mKRAB-1 (5'-GCTGCGGCTGTGCTCTTTACTCGGGACGAG-3') and mKRAB-2 (5'-TTCAAATGTCACTGACACTGCTAG-3'), and templates pFB-tTS-Neo and pFB-tTS-EYFP-Neo.

TetR-fusion protein genes and the neomycin resistance gene were cloned into a retrovirus vector bearing an internal ribosome entry sequence (IRES). Virus-infected cells were maintained in medium containing neomycin and/or 1 μg/ml of doxycycline.

Microscopy and Image Analysis

Cells were transfected, fixed for 10 min with 4% PFA and mounted with VectaShied for microscopy, which was performed with a DeltaVision (Applied Precision, Issequah, WA) inverted microscope. For analysis of the intensity of various FPs on the HAC, Z-stacks were acquired using the same Z-spacing and exposure without deconvolution. We then defined a cylindrical region of interest through the stacks using the image analysis tool of Softworx and summed the intensity within this region for each image plane. The total intensity for image planes containing the HAC is shown in Fig. 4B. This was normalized for background by division by the summed intensity above and below the HAC within the cylindrical ROI.

ImmunoFISH and Cytological Preparations

ImmunoFISH experiments were performed on chromosome spreads. Cells from alphoid^{tetO} HAC cell line AB2.2.18 were incubated for 4 hours in 0.1 μg/ml colcemid and after mitotic shake-off, mitotic cells were resuspended in 75 mM KCl at 37°C and incubated for 10 min. After centrifugation, mitotic cells

were fixed for 15 min with cold (-20 °C) MeOH. Mitotic spreads were transferred by dropping onto a clean glass slide, dried and incubated in PBST (1x PBS + 0.05% Tween 20) for 5 min. After pre-block with 1% BSA in PBST for 30 min at 37 °C, incubation with primary antibody was done o/n at 4 °C and with secondary for 30 min at 37 °C. After the second incubation, cells were fixed again with 4% PFA for 8 min, washed twice with 2x SSC buffer for 5 min and EtOH dehydrated. Another wash was done with 2x SSC for 45 min at increasing temperature from 25 to 70 °C, followed by EtOH dehydration. DNA was denaturated with 0.1 M NaOH for 10 min, the slides were washed 3 times with 2x SSC and dehydrated. BAC-probe (obtained using Bionick Labelling kit from Invitrogen) was denaturated and applied to the slides, which were incubated for 2 min on a thermoblock at 75 °C. Hybridization was o/n at 39 °C in a humidified chamber. The following day slides were washed 3 times for 5 min with 2x SSC at 45 °C and again for 5 min at room temperature. Incubation with FITC-avidin (Molecular Probes, Inc.) 1:500 for 30 min at 37 °C was followed by incubation with biotinylated anti-avidin antibody (Vector) (1:100) for 30 min at 37°C and by another final incubation with FITC-avidin. Slides were finally washed for 5 min with 2x SSC and mounted with VectaShield (Vector). Chromatin fibers were obtained from alphoid^{tetO} HAC cell line AB2.2.18.21 and processed for ImmunoFISH as described (Blower et al., 2002).

Chromatin Immunoprecipitation (ChIP) and Real-Time PCR

ChIP with CENP-B antibody (2D8D8 and 5E6C1) was carried out according to a previously described method (Nakano et al., 2003). ChIP with antibody

against EYFP (anti-Green Fluorescent Protein, Roche) was done using a modified method. Cultured cells were cross-linked in 1.0% formaldehyde. Soluble chromatin was prepared in sonication buffer (5 mM HEPES, 1.5 μM aprotinin, 10 μM leupeptin, 1 mM DTT, 0.5% SDS and 40 μM MG132) and immunoprecipitated in IP buffer (30 mM HEPES, 150 mM NaCl, 1 mM EGTA, 2 mM $MgCl_2$, 2 mM ATP, 1.5 μM aprotinin, 10 μM leupeptin, 1 mM DTT, 0.05% SDS, 1% Triton X-100). ChIP with antibodies against CENP-A (mAN1), dimethyl histone H3 Lys4 (Upstate), trimethyl H3 Lys4 (Upstate) and trimethyl H3 Lys9 (Upstate) was done by another modified method. Cultured cells were cross-linked in 0.5% formaldehyde for 5 min, washed with TBS buffer (25 mM Tris-Cl, 137 mM NaCl, 2 mM KCl, pH 7.4), frozen in liquid nitrogen and stored at -80ºC until use. Soluble chromatin was prepared by sonication (Bioruptor sonicator, Cosmo Bio) to an average DNA size of 0.5 kb in sonication buffer (20 mM Tris, pH 8.0, 1 mM EDTA, 1.5 μM aprotinin, 10 μM leupeptin, 1 mM DTT and 40 μM MG132), and immunoprecipitated in IP buffer (20 mM Tris-HCl, pH 8.0, 600 mM NaCl, 1 mM EDTA, 0.05% SDS, 1.0% TritionX-100, 20% glycerol, 1.5 μM aprotinin, 10 μM leupeptin, 1 mM DTT and 40 μM MG132). Protein G Sepharose (Amersham) blocked with bovine serum albumin was added, and the antibody-chromatin complex was recovered by centrifugation.

The recovery ratio of the immunoprecipitated DNA from input DNA was measured by real-time PCR using the following primer sets: 5SDNA-F1 and 5SDNA-R1 for 5S ribosomal DNA, 11-10R and mCbox-4 for 11-mer of chromosome 21 alphoid DNA (alphoid$^{chr. 21}$) (Nakano et al., 2003), Sat2-F1 and Sat2-R1 for pericentromeric satellite 2 repeat (Nakashima et al., 2005),

tet-1 (5’-CCACTCCCTATCAGTGATAGAGAA-3’) and tet-3 (5’-TCGACTTCTGTTTAGTTCTGTGCG-3’) for the alphoid^{tetO} HAC, SA3 and JRN (5’- AATTCACTAGCGAATTCCC-3’) for cloned alphoid DNA of a control HAC derived from synthetic alphoid 11-mer (alphoid^{11mer}) (Ohzeki et al., 2002), bsr-F and bsr-R for the marker gene (*bsr*) of alphoid^{tetO} HAC (Nakano et al., 2003), NEO1 (5’-TGGATTGCACGCAGGTTCTCCGGC-3’) and NEO2 (5’-GGCATCAGAGCAGCCGATTGTCTG-3’) for the marker gene (*Neo^r*) of the wt11-mer HAC.

The Analysis by Real-Time PCR of the HAC Loss Rate (R) in Subclones Expressing the tTA

Genomic DNAs of mRFP-tetR or tTA expressing subclones were purified and processed as described above. Additional primer sets included, 17alpF (5’-CAACTCCCAGAGTTTCACATTGC-3’) and 17alpR (5’-GGAAACTGCTCTTTGAAAAGGAACC-3’) for chromosome 17 alphoid DNA, X3-F (5’-GTGACGATGGAGTTTAACTCAGGG-3’) and X4-R (5’-GCTTTCCGTTCAGTTATGGGAAGG-3’) for chromosome X alphoid DNA.

Indirect Immunofluorescence

Indirect immunofluorescence was carried out as previously described. Cells expressing EYFP-TetR or EYFP-tTS were cultured on poly-D-Lysine-coated coverslips, fixed in 1% formaldehyde for 10 min, treated with methanol for 5 min and dried. The coverslips were then treated with 0.5% Triton X-100 and 0.1 M glycine for 5 min each. Antibodies used were anti-CENP-A (mAN1), anti CENP-C (Ra1), anti-GFP monoclonal antibody (Invitrogen), and/or anti-GFP polyclonal antibody (Medical & Biological Laboratories co., ltd, Japan). Images were captured using a Zeiss microscope (Axiophot) equipped with a cooled-

charge-coupled device (CCD) camera (Cool SNAP HQ, Photometrics) and analyzed by IPLab software (Signal Analytics).

Quantification of Transcripts Derived from alphoid^{tetO} HAC

Real-time RT-PCR was carried out using the iScript One-Step RT-PCR Kit with SYBR Green (Bio-Rad) according to the manufacturer's protocol, using total RNA prepared with the SV Total RNA Isolation system (Promega). Reverse transcription and PCR were done with the following primer sets: hActin-a (5'-ATCTGGCACCACACCTTCTACAATGAGCTGCG-3') and hActin-b (5'-CGTCATACTCCTGCTTGCTGATCCACATCTGC-3') for human *β-actin*, 11-10R and mCbox-4 for 11-mer of chromosome 21 alphoid DNA (alphoid$^{chr. 21}$), tet-1 and tet-3 for the alphoid^{tetO}. bsr-F and bsr-R for the marker gene (*bsr*) of alphoid^{tetO} HAC.

Methylated DNA Immunoprecipitation (MeDIP)

MeDIP with anti 5-meC antibody (Diagenode) was carried out using a modified method. Purified genomic DNA was sheared using a Bioruptor sonicator (Cosmo Bio) to an average DNA size of 0.5 kb and denatured at 94˚C for 10 minutes. The methylated DNA was immunoprecipitated in IP buffer (20 mM Tris, 150 mM NaCl, 1 mM EDTA, 20% glycerol, 1.5 μM aprotinin, 10 μM leupeptin, 1 mM DTT, 0.1% NP-40). Protein A agarose blocked with salmon sperm DNA (Upstate) was added, and the antibody-DNA complex was recovered by centrifugation. The recovery ratio of the immunoprecipitated DNA from input DNA was measured by real-time PCR using the following primer sets: 11-10R and mCbox-4 for 11-mer of

chromosome 21 alphoid DNA (alphoid$^{chr.\ 21}$), tet-1 and tet-3 for the alphoid^{tetO}. bsr-F and bsr-R for the marker gene (*bsr*) of alphoid^{tetO} HAC.

Supplemental References

Agata, Y., Matsuda, E., and Shimizu, A. (1999). Two novel Kruppel-associated box-containing zinc-finger proteins, KRAZ1 and KRAZ2, repress transcription through functional interaction with the corepressor KAP-1 (TIF1beta/KRIP-1). J. Biol. Chem. *274*, 16412-16422.

Blower, M. D., Sullivan, B. A., and Karpen, G. H. (2002). Conserved organization of centromeric chromatin in flies and humans. Dev. Cell *2*, 319-330.

Masumoto, H., Sigimoto, K., and Okazaki, T. (1989). Alphoid satellite DNA is tightly associated with centromere antigens in human chromosomes throughout the cell cycle. Exp. Cell. Res. *181*, 181-196.

Matsuda, E., Agata, Y., Sugai, M., Katakai, T., Gonda, H., and Shimizu, A. (2001). Targeting of Kruppel-associated box-containing zinc finger proteins to centromeric heterochromatin. Implication for the gene silencing mechanisms. J. Biol. Chem. *276*, 14222-14229.

Nakano, M., Okamoto, Y., Ohzeki, J., and Masumoto, H. (2003). Epigenetic assembly of centromeric chromatin at ectopic alpha-satellite sites on human chromosomes. J. Cell Sci. *116*, 4021-4034.

Nakashima, H., Nakano, M., Ohnishi, R., Hiraoka, Y., Kaneda, Y., Sugino, A., and Masumoto, H. (2005). Assembly of additional heterochromatin distinct from centromere-kinetochore chromatin is required for de novo formation of human artificial chromosome. J. Cell Sci. *118*, 5885-5898.

Ohzeki, J., Nakano, M., Okada, T., and Masumoto, H. (2002). CENP-B box is required for de novo centromere chromatin assembly on human alphoid DNA. J. Cell Biol. *159*, 765-775.

Table S1A. Efficiency of HAC Formation Following Transfection with alphoid BACs

Introduced DNA	Analyzed cell lines	No. of cell lines : either HAC or integration signals	
		HAC	Host chromosome integration
wild type 11.32	41	12 (29.3%)	29 (70.7%)
BAC32-2mer(tetO)	46	2 (4.3%)	40 (87.0%) *

* 4 (8.7%) cell lines showed extra minichromosome signals containing host chromosomal fragments.

Table S1B. Characterization of BAC32-2mer(tetO) Derived HACs

clone	loss rate	copy number of either BAC32-2mer(tetO)		ratio of cells: HAC or integration signals		
					integration into host	
		$alphoid^{tetO}$	bsr	HAC	centromere	arm
AB2.2.18				35.7%	0	64.3%
AB2.2.18.21*	0.0024	47.6	46.5	100%	0	0
AB2.5.4				28.6%	71.4%	0
AB2.5.4.19**	0.0054	16.8	15.8	100%	0	0

* subclone of AB2.2.18

** subclone of AB2.5.4

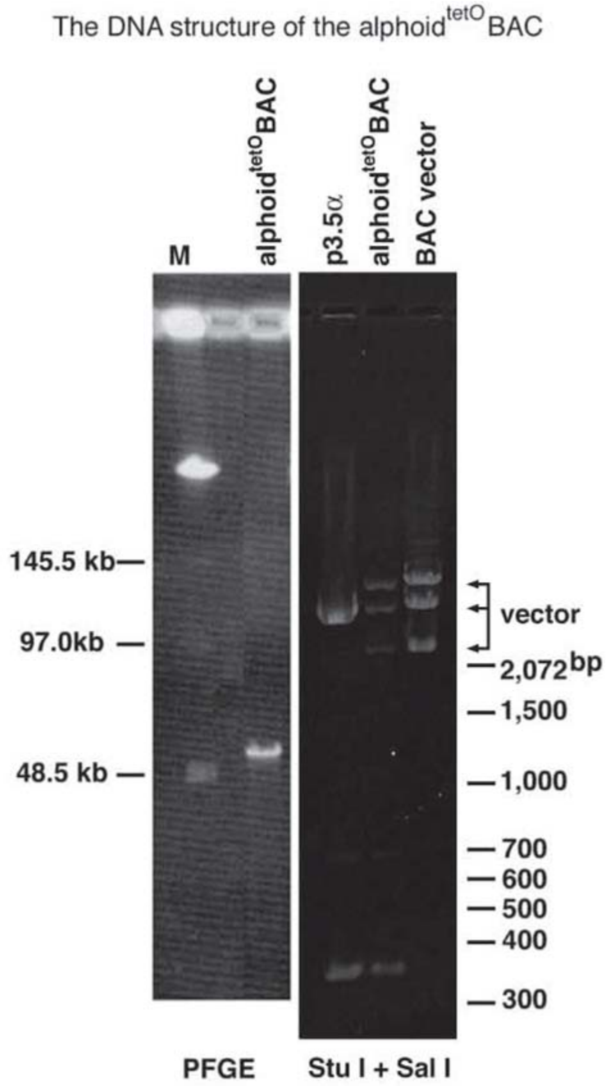

Figure S1. The alphoid^{tetO} BAC Contains an Unrearranged Head-to-Tail Array of alphoid^{tetO} Dimer

(left panel) Ethidium bromide stained gel following PFGE of purified alphoid^{tetO} BAC [BAC32-2mer(tetO)] treated with restriction enzyme *NotI*. (right panel) Structural analysis of alphoid^{tetO} BAC with the restriction enzyme *StuI* and *SalI*. Alphoid^{tetO} BAC and p3.5α (a plasmid containing 10 repeats of alphoid^{tetO} dimer) were treated with *StuI* whose restriction site appears once per alphoid^{tetO} dimer. Alphoid^{tetO} BAC was simultaneously treated with *StuI* and *SalI* appears at alphoid^{tetO} dimer cloning site, and was compared with empty BAC vector.

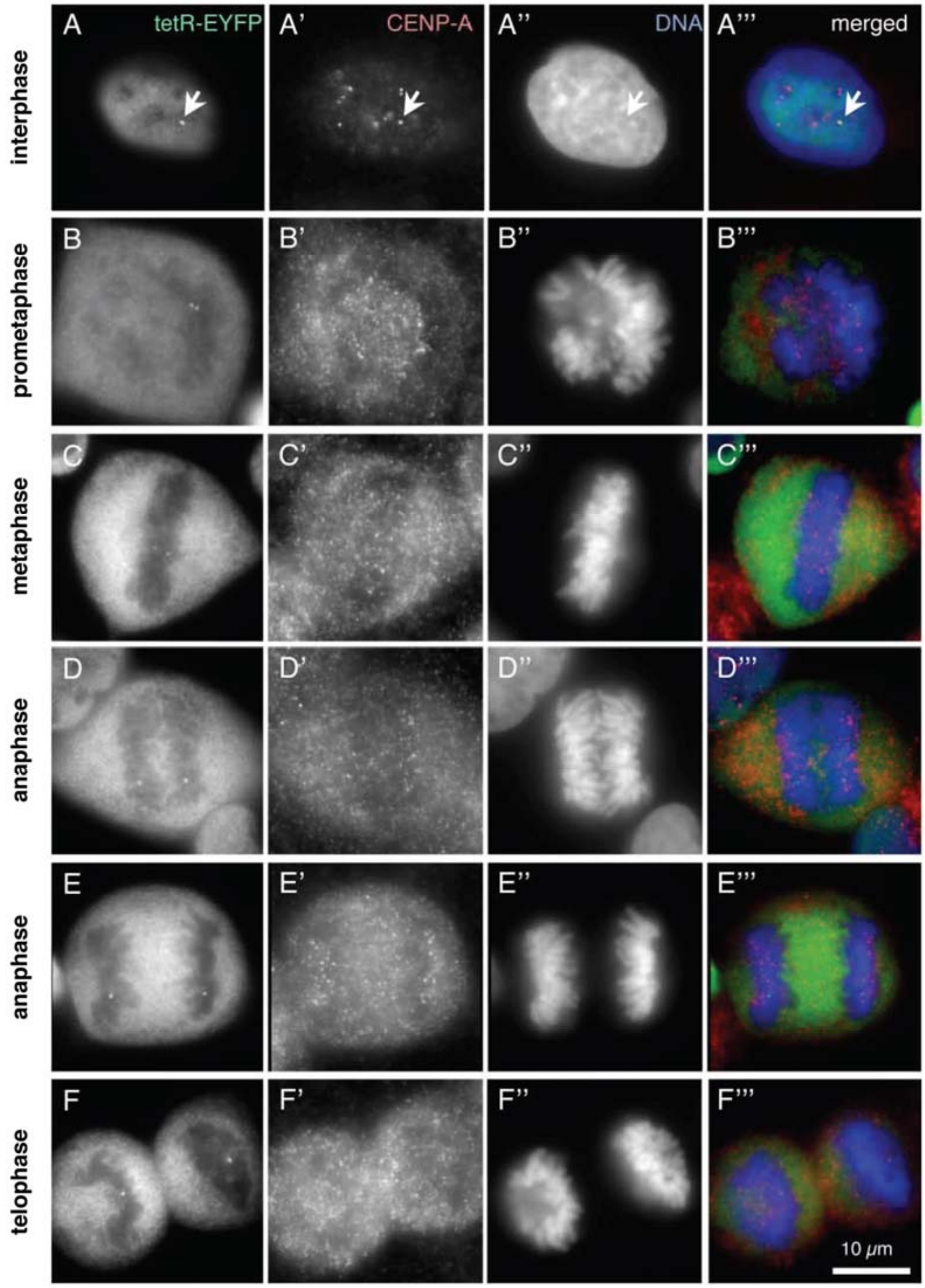

Figure S2. The alphoid^{tetO} HAC Targeted by tetR-EYFP Can Segregate Correctly during Cell Division

Alphoid^{tetO} HAC cell line (AB2.2.18.21) stably expressing tetR-EYFP was immunostained with anti-GFP (green, A-F) and anti-CENP-A (red, A'-F') antibodies. Chromosomes were stained with DAPI (blue, A"-F"). In interphase, tetR-EYFP and CENP-A colocalized to the alphoid^{tetO} HAC (A). The alphoid^{tetO} HAC was observed as a pair of dots in prometaphase (B) and aligned at metaphase plate at metaphase (C). In anaphase, sister chromatids of alphoid^{tetO} HAC were separated and moved toward each spindle pole (D and E) and segregated to daughter nuclei (F). Arrows indicate the alphoid^{tetO} HACs. Size bar = 10 μm.

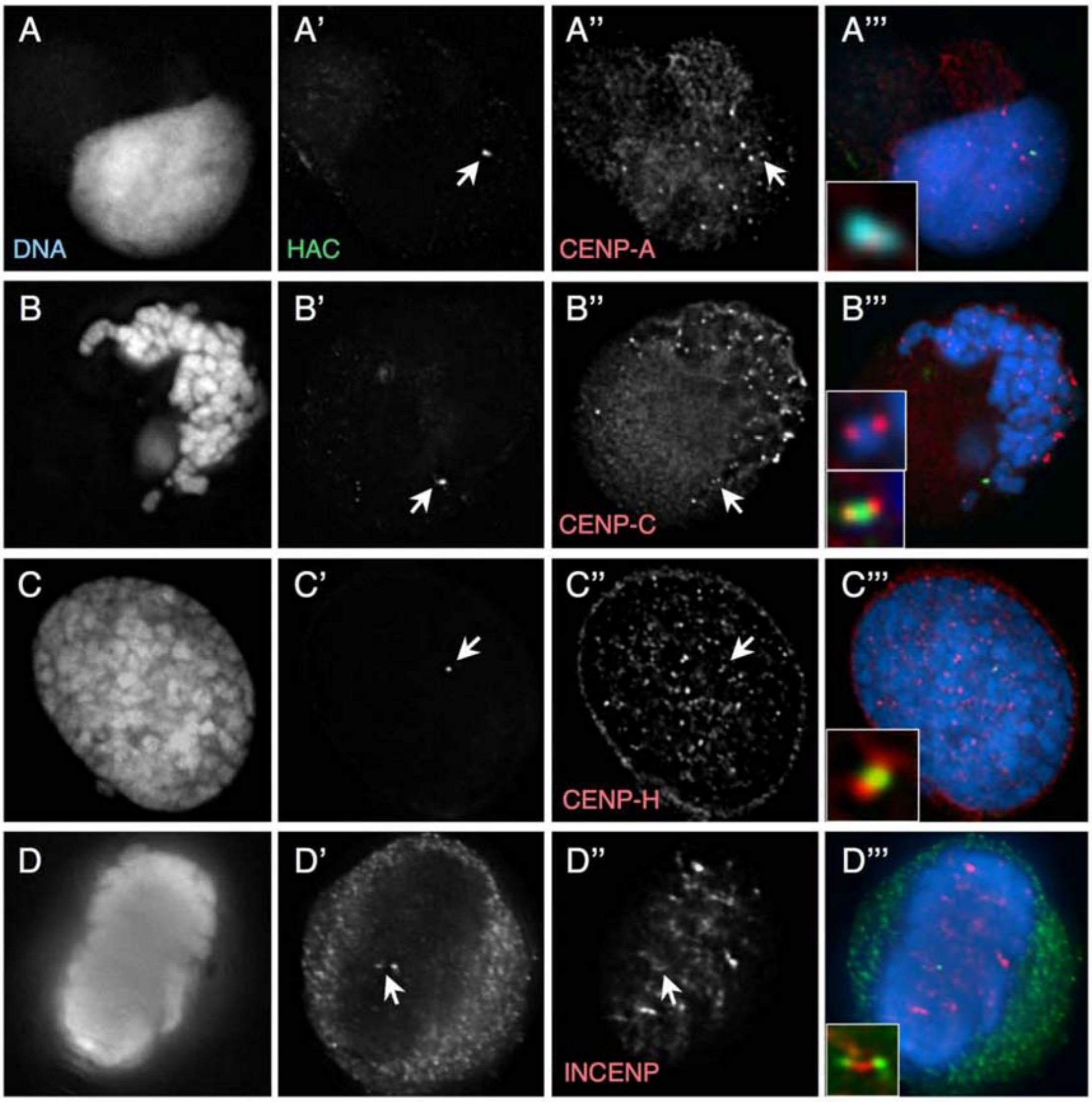

Figure S3. The alphoid^{tetO} HAC Recruits Several Centromere/Kinetochore Proteins throughout the Cell Cycle

The alphoid^{tetO} HAC (arrows) was identified by FISH with a BAC probe (green, A'-D'), and shown to colocalize with CENP-A (red, A''), CENP-C (red, B''), CENP-H (red, C'') and INCENP (red, D''). Insets show the colocalization of the HAC and kinetochore proteins by immunoFISH. The cells shown were in interphase (panels A), prometaphase (panels B), prophase (panels C) and metaphase (panels D). Arrows indicate HACs.

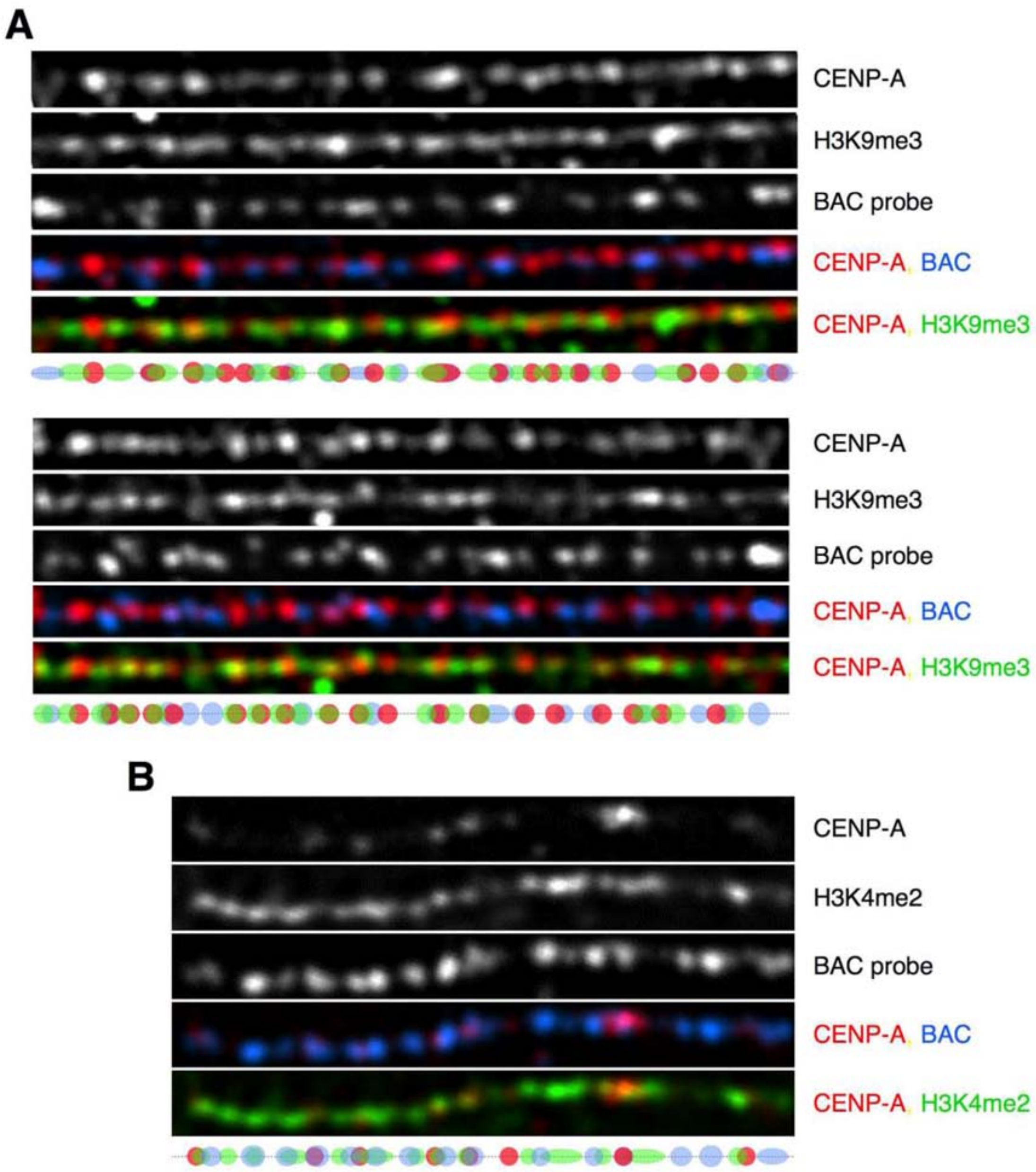

Figure S4. CENP-A Localizes Predominantly in alphoid Regions along the Length of the HAC

ImmunoFISH was performed on chromatin fibers obtained from the alphoid^{tetO} HAC cell line (AB2.2.18.21), using antibodies for CENP-A, H3K9me3, H3K4me2 (Upstate) and a BAC-probe. CENP-A localizes predominantly in the alphoid^{tetO} regions between BAC-positive regions along the length of the fibers (red in panel A and B). Histone modifications H3K9me3 and H3K4me2 (green) are also present on the alphoid^{tetO} array, flanking CENP-A positive regions. They also partially overlap with BAC regions, particularly H3K4me2 (B).

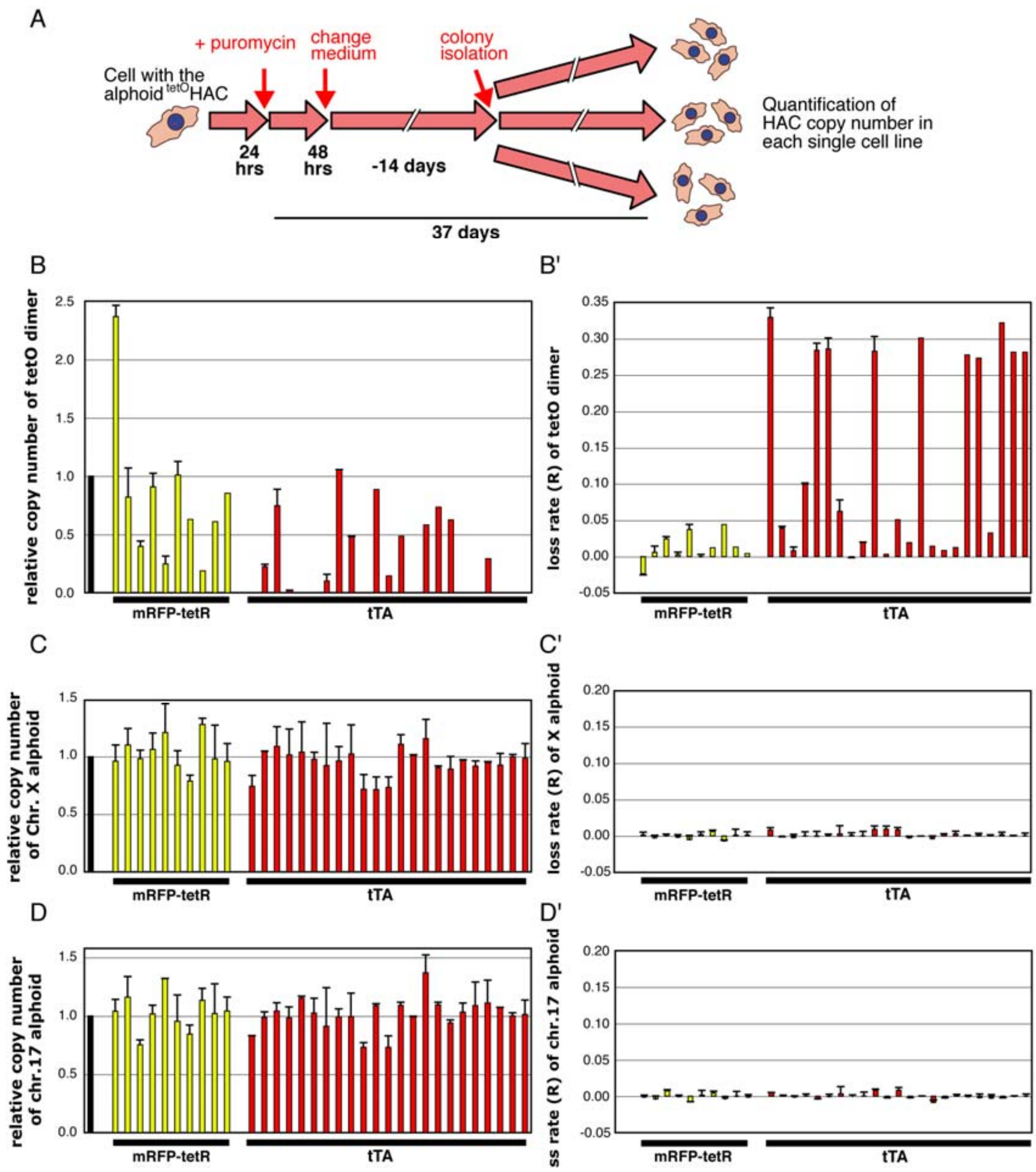

Figure S5. Real-Time PCR Colony Assay for tTA Induced Instability of the alphoid^{tetO} HAC

(A) A summary of sub-cell line analysis. RFP-tetR and tTA expression plasmids were transfected to the alphoid^{tetO} HAC cell. After 37 days, copy numbers of the HAC in individual sub-cell lines were quantified by real-time PCR. (B) The relative copy numbers of alphoid^{tetO} dimer DNA in HAC sub-cell line AB2.5.4.19 transfected with the tTA (red bars - 23 separate colonies) or mRFP-tetR (yellow bars - 10 colonies) were analyzed by real-time PCR and compared with the level in the original cell line (black bar). Colonies were cultured in non-selective media for 37 days. (B') Chromosome loss rate of the alphoid^{tetO} HAC per generation of the colonies was calculated using the formula [$N_{37} = (1-R)^{37}$, where N_{37} = relative copy number of the alphoid^{tetO} DNA after 37 generations]. Significant loss of the alphoid^{tetO} HAC was observed in 10 colonies (43.5%) of tTA expressing cell lines ($P < 0.01$). The relative copy numbers of endogenous host chromosome X (C) and 17 (D) alphoid DNA were also measured. Expression of tTA induced no increased loss rate of these host alphoid DNAs relative to that seen in cells expressing mRFP-tetR (C' and D') ($P > 0.15$ or 0.83). Error bars indicate s.d. For statistical analysis, the Student's t-test was applied.

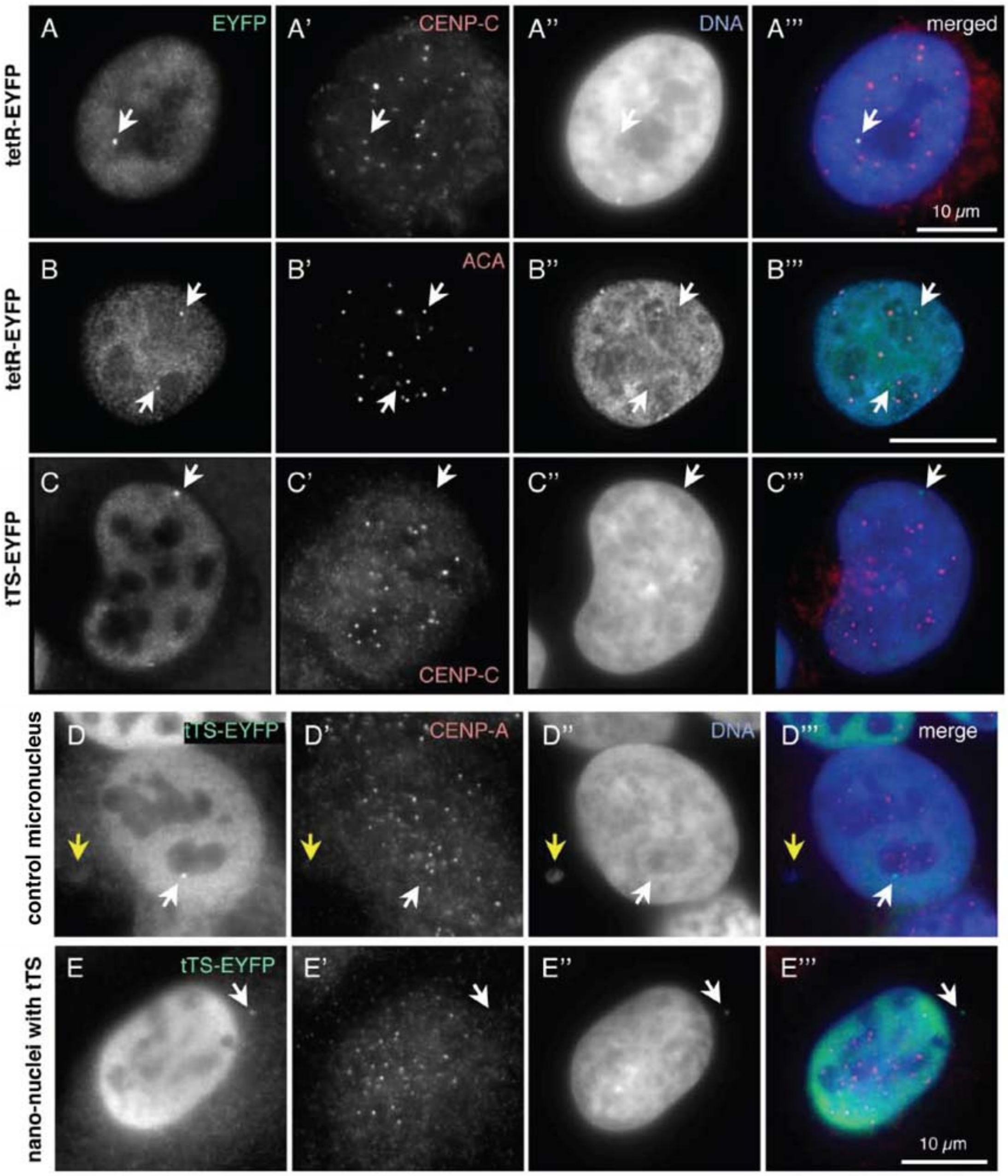

Figure S6. tTS Targeting Induces Disassembly of CENP-A, -B and -C from the alphoid^{tetO} HAC and Causes Formation of Nano-Nuclei

Alphoid^{tetO} HAC cell lines expressing tetR-EYFP (A and B) or tTS-EYFP (C-E) were analyzed by indirect immunofluorescence using anti-GFP (green, A-E) and anti-CENP-C (red, A' and C'), ACA (red, B') or anti-CENP-A (D' and E') antibodies. DNA was stained by DAPI (blue, A"-E"). The tetR-EYFP (A and B) colocalized with CENP-C (A') ACA antigen (B') on the alphoid^{tetO} HAC. On HACs targeted by tTS-EYFP, CENP-C assembly was not detected (C'). Nano-nuclei were observed in HAC cell lines expressing the tTS (D and E). The HAC are indicated by white arrows. Yellow arrows indicate micronuclei derived from endogenous chromosomes. Size bars = 10 μm.

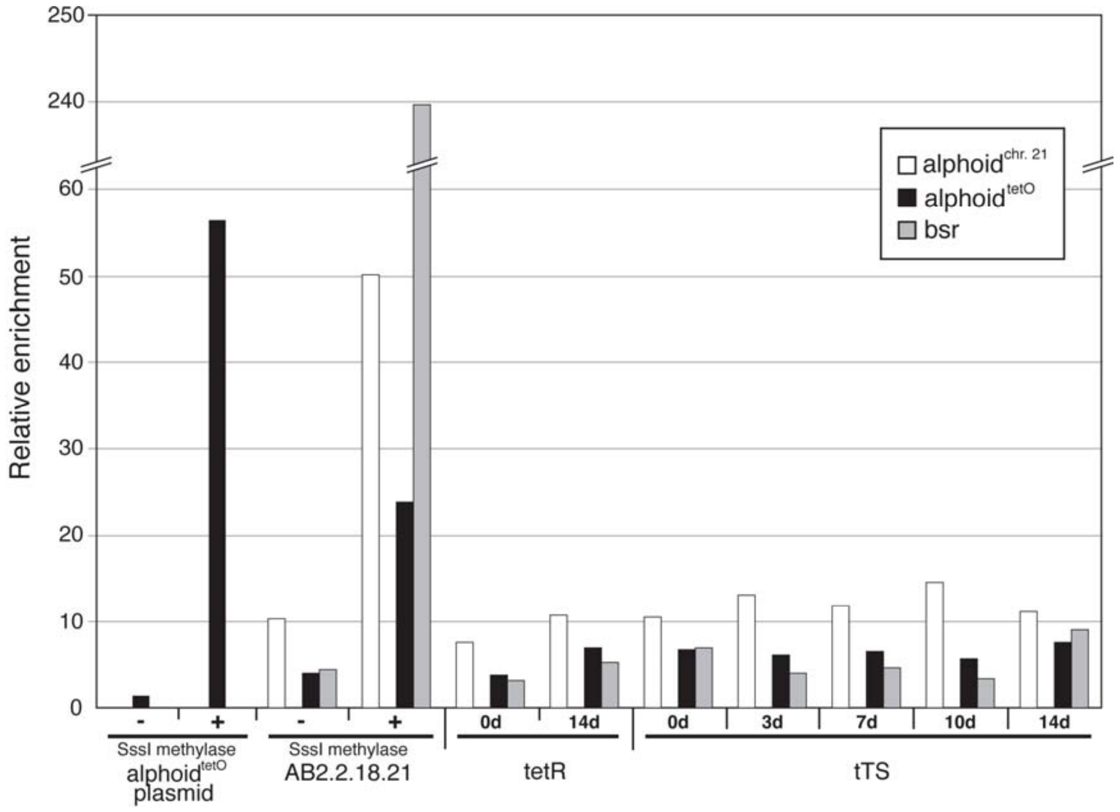

Figure S7. CpG Methlation of alphoid^{tetO} HAC DNA Analyzed by Methylated DNA Immunoprecipitation (MeDIP) with Anti 5-Methyl Cytidine Antibody

Genomic DNA was purified from alphoid^{tetO} HAC cells (AB2.2.18.21) expressing tetR or tTS cultured in doxycycline free medium for 3, 7, 10 and 14 days. The purified DNA was immunoprecipitated with antibody against 5-methylated Cytidine and quantitated by real-time PCR. As controls, the genomic DNA of the original cell (AB2.2.18.21) and purified alphoid^{tetO} dimer plasmid DNA (p3.5α) were treated with or without Sss I CpG methylase in vitro and were analyzed by MeDIP assay. The bars show the relative enrichment of alphoid$^{chr. 21}$ (white), alphoid^{tetO} (black) and the *bsr* gene (gray). The level of the CpG methylated alphoid^{tetO} HAC DNA did not increase drastically during 14 days of culture even after tTS binding.
